# A Comprehensive Review of AI Diagnosis Strategies for Age-Related Macular Degeneration (AMD)

**DOI:** 10.3390/bioengineering11070711

**Published:** 2024-07-13

**Authors:** Aya A. Abd El-Khalek, Hossam Magdy Balaha, Ashraf Sewelam, Mohammed Ghazal, Abeer T. Khalil, Mohy Eldin A. Abo-Elsoud, Ayman El-Baz

**Affiliations:** 1 Communications and Electronics Engineering Department, Nile Higher Institute for Engineering and Technology, Mansoura 35511, Egypt; ayayakot94@nilehi.edu.eg; 2 Department of Bioengineering, J.B. Speed School of Engineering, University of Louisville, Louisville, KY 40292, USA; 3 Ophthalmology Department, Faculty of Medicine, Mansoura University, Mansoura 35511, Egypt; a_sewelam_eg@mans.edu.eg; 4 Electrical, Computer, and Biomedical Engineering Department, Abu Dhabi University, Abu Dhabi 59911, United Arab Emirates; mohammed.ghazal@adu.ac.ae; 5 Communications and Electronics Engineering Department, Faculty of Engineering, Mansoura University, Mansoura 35511, Egypt; abeer.tawkol@mans.edu.eg (A.T.K.); mohyldin@mans.edu.eg (M.E.A.A.-E.)

**Keywords:** age-related macular degeneration (AMD), deep learning (DL), machine learning (ML), retinal segmentation, retinal disease diagnosis

## Abstract

The rapid advancement of computational infrastructure has led to unprecedented growth in machine learning, deep learning, and computer vision, fundamentally transforming the analysis of retinal images. By utilizing a wide array of visual cues extracted from retinal fundus images, sophisticated artificial intelligence models have been developed to diagnose various retinal disorders. This paper concentrates on the detection of Age-Related Macular Degeneration (AMD), a significant retinal condition, by offering an exhaustive examination of recent machine learning and deep learning methodologies. Additionally, it discusses potential obstacles and constraints associated with implementing this technology in the field of ophthalmology. Through a systematic review, this research aims to assess the efficacy of machine learning and deep learning techniques in discerning AMD from different modalities as they have shown promise in the field of AMD and retinal disorders diagnosis. Organized around prevalent datasets and imaging techniques, the paper initially outlines assessment criteria, image preprocessing methodologies, and learning frameworks before conducting a thorough investigation of diverse approaches for AMD detection. Drawing insights from the analysis of more than 30 selected studies, the conclusion underscores current research trajectories, major challenges, and future prospects in AMD diagnosis, providing a valuable resource for both scholars and practitioners in the domain.

## 1. Introduction

The initial definition of age-related macular degeneration (AMD), described as symmetric central chorioretinopathy in the elderly, was in 1874. AMD is a chronic ocular disease that impairs the eye’s central vision. The macula, the core region of the retina responsible for sharp, clear vision, is degenerating [1,2]. AMD now accounts for 8.7% of blindness worldwide and is one of the leading causes of permanent vision loss in the elderly (>50 years old). In Europe, approximately 1% of people have advanced AMD. It is projected that 288 million people worldwide will suffer from AMD by 2040. In addition to aging, hereditary and environmental variables are the primary causes of AMD [3,4].

Eye diseases are a major health concern for the elderly. People who have eye disorders usually do not realize their symptoms are slowly getting worse. Routine eye exams are, therefore, necessary for an early diagnosis. For prompt intervention, AMD must be accurately diagnosed and detected as early as possible. Typically, a slit-lamp examination is used by an ophthalmologist to diagnose eye conditions [5]. Due to variations in the ophthalmologist’s analytical abilities, irregularities in the analysis of eye disorders, and problems with record keeping, slit-lamp interpretations are insufficient. Fundus images, captured through retinal imaging, provide valuable information about the structural changes in the macula associated with AMD [6].

Analyzing images from Optical Coherence Tomography (OCT), OCT Angiography, and fluorescein angiography (FA), or fundus cameras is known as retinal image analysis. These predominant non-invasive methods for capturing alterations in retinal structure, encompassing changes in the optic disc, blood vessels, macula, and fovea, are fundoscopy and OCT imaging. Analyzing these images assists in identifying various conditions such as diabetic retinopathy (DR), cataracts, glaucoma, AMD, myopia, and high blood pressure [7].

AMD fundus image classification into distinct categories plays a vital role in understanding the disease progression and determining appropriate treatment plans. As shown in Table 1, Four phases are commonly used to classify AMD: normal, early, intermediate, and advanced. No or early-stage AMD is diagnosed when there are either no Drusen or only a few small Drusen (macular yellow deposits) present in the eye. In early AMD, Drusen are considered small to medium in size. Intermediate AMD is characterized by numerous medium-sized Drusen or at least one large Drusen, sometimes accompanied by pigmentary changes. Advanced AMD can manifest in either dry or wet forms. Dry AMD occurs when the retinal pigment epithelium near the macula degenerates, leading to Geographic Atrophy (GA). This atrophic area gradually progresses toward the center of the macula, resulting in permanent vision loss if it involves the central region. On the other hand, wet AMD progresses rapidly with a sudden onset. It is caused by abnormal growth of blood vessels behind the retina, which can rupture or leak, causing swift deterioration of vision [8].

Computer-aided diagnostic (CAD) systems leverage advancements in machine learning (ML) and computer vision to diagnose conditions like DR and AMD. ML techniques exhibit significant potential in accurately categorizing AMD by identifying subtle characteristics indicative of various disease stages [9]. These methods automate the classification process, ensuring consistent and objective assessments while reducing variability among observers and facilitating timely diagnoses [10,11]. Despite the challenges involved, such as the need for extensive effort in feature extraction, analysis, and engineering, as well as a deep understanding of disease-specific traits, ML-inspired approaches have shown promising outcomes [12]. Furthermore, the emergence of deep learning (DL) as a subfield of ML has shown promise in detecting specific retinal illnesses. DL techniques have significantly advanced the identification and classification of ocular diseases such as Diabetic Macular Edema (DME), AMD, and DR. Transformer networks, initially popularized in natural language processing, are being adapted for computer vision tasks, particularly in retinal disease diagnosis [13,14]. The development of a reliable system for classifying Optical Coherence Tomography and fundus images holds great promise for clinical application. Such a system could assist ophthalmologists in guiding patients toward appropriate treatment plans [15].

**Figure 1:**
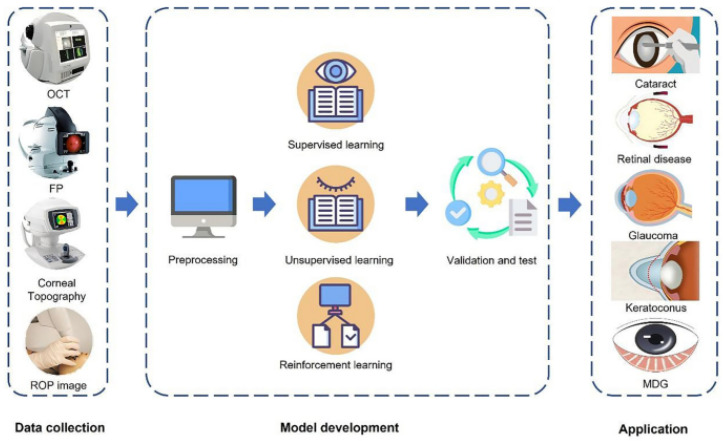
AI workflow in ophthalmology usually includes model building, training, validation, testing, and implementation in addition to data gathering and preprocessing. It is intended to use deep learning and machine learning methods to diagnose, treat, and manage eye conditions [17].

In medicine, artificial intelligence (AI) has been extensively used in a variety of settings Figure 1. Particularly in the field of ophthalmology, partnerships between medical imaging and AI disciplines have shown to be particularly fruitful. Professional ophthalmologists currently cannot afford to do such a high volume of AMD screening, particularly in impoverished nations and areas. Three areas of AI technology for AMD diagnosis exist (1) Disease/no disease, (2) wet/dry/no disease, and (3) AMD severity. With AI’s help, AMD may reduce the time it takes to diagnose and treat patients, enhance the effectiveness of diagnosis and therapy, and delay the onset of AMD blindness, which addresses a flaw in many medical businesses [16].

The primary goal of the proposed review is to give a thorough analysis of the different ML and DL techniques that have been recently used for AMD using different modalities. The proposed review discusses the methodology employed, encompassing data collection, pre-processing techniques, feature extraction methods, and the selection of ML algorithms. The contributions can be summarized as follows:

- It discusses the findings, challenges, advantages, disadvantages, and limitations of ML and DL-based AMD classification systems, offering insights for future research directions in AI-based AMD diagnosis.
- It utilizes the DL process pipeline technique for AMD retinal disease diagnosis, evaluating recent papers on AMD and providing information on DL backbone models, assessment measures, and image pre-processing methods.
- It includes a tabulated analysis of comparative performances of various DL implementations, a thorough literature study, a discussion on the utilization of public and private datasets, and outlines current avenues of research, contributing to the advancement of computer-aided diagnosis of AMD and enhancing patient care and outcomes.

The structure of this review is organized as follows: Section 2 presents prevalent imaging modalities. Section 3 outlines the AMD diagnosing framework with its internal three main components: AMD Datasets Acquisition, Pre-Processing Techniques, and Deep and Machine Learning Techniques for AMD Segmentation and Diagnoses. Section 4 presents the Discussion and Summary of this research. Section 5 presents the future research directions. Finally, Section 6 concludes the review.

## 2. Prevalent Imaging Modalities

In exploring the complexities of the human eye, various imaging methods have been developed such as Color Fundus Photographs (CFP), OCT, OCT Angiography (OCTA), Fluorescein Angiography (FA), and Indocyanine Green Angiography (ICG), each with its unique role in understanding ocular health, in addition, each imaging modality is better suited for the type of disease that doctors are dealing with and to interpret the findings [18].

This section discusses various imaging methods used in AMD and retinal disorders diagnosis and focuses on the different modalities techniques that have demonstrated potential in treating AMD and retinal diseases diagnostics because they are helpful in the identification, monitoring, counseling, diagnosis, and prognosis of several ocular illnesses according to [2,10,19,20,21,22,23], including glaucoma, DR, ARMD, and retinal vascular disorders.

**Figure 2:**
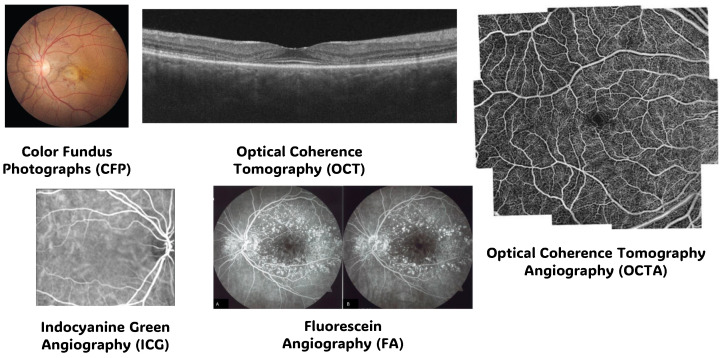
Samples from the prevalent imaging modalities: Color Fundus Photographs (CFP), Optical Coherence Tomography (OCT), OCT Angiography (OCTA), Fluorescein Angiography (FA), and Indocyanine Green Angiography (ICG) [26,27,28,29,30].

CFP provides detailed images of the back of the eye, capturing the retina, optic nerve, and blood vessels, aiding in the diagnosis and monitoring of various eye conditions such as macular degeneration and diabetic retinopathy. OCT utilizes light waves to produce high-resolution cross-sectional images of the retina, allowing for precise assessment of its layers and identifying abnormalities like macular edema and retinal thinning. OCTA enhances OCT by visualizing blood flow within the retina and choroid without the need for dye injection, facilitating the detection of vascular irregularities like neovascularization and microaneurysms. FA involves injecting a fluorescent dye into the bloodstream to capture detailed images of the retinal vasculature, helping diagnose conditions such as macular edema, retinal vein occlusion, and choroidal neovascularization (CNV). ICG employs a different dye and longer wavelength light to visualize deeper choroidal vessels, aiding in the assessment of conditions like choroidal tumors, polyps, and inflammatory disorders. Each imaging modality offers unique insights into ocular health, allowing for comprehensive evaluation and management of retinal diseases. Sample from each modality is presented in Figure 2 [18,24,25,26,27,28,29,30].

### 2.1. Color Fundus Photographs (CFP) Modality

**Figure 3:**
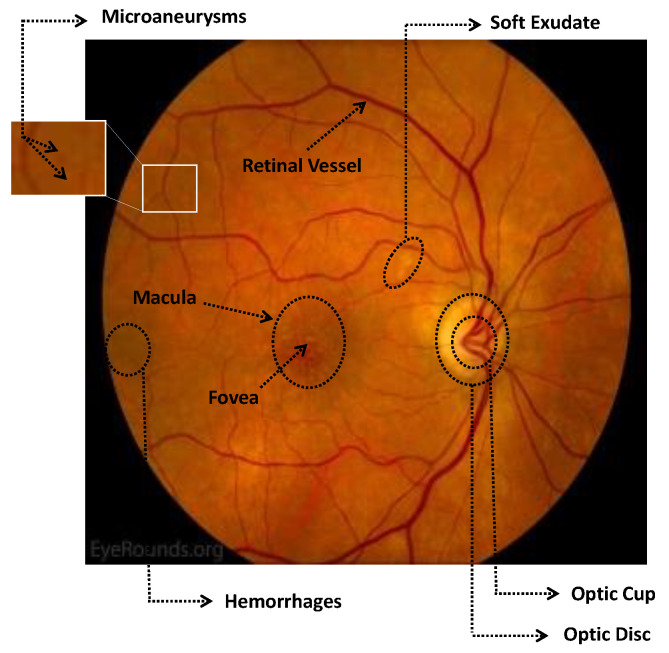
A fundus image with pathologies that presents the microaneurysms, hemorrhages, and fovea.

Numerous imaging methods have been created over the years to study the human eye; among them, “Fundus Imaging” has become more well-known because it is non-invasive and reasonably priced. When screening for retinal abnormalities, color fundus photography is the most economical imaging technique to use. The technique of utilizing a monocular camera to project the fundus, or the back of an eye, onto a two-dimensional plane is known as fundus photography. Many ocular structures and biomarkers, including various abnormalities, can be identified from a recorded 2D fundus image. Numerous of these visual indicators are crucial in the diagnosis of retinal disorders [31]. Figure 3 displays a fundus image with pathologies.

The fundus image, which is often captured by image sensors in three colors, is a reflection of the eye’s inside surface. It contains details on the biological features something the unaided eye can see, such as the macula, optic disc, retinal vasculature, and retinal surface. Because blue light is absorbed by the posterior retinal pigment layer and blood vessels, their spectral range makes the anterior retinal layers more visible. Concurrently, the pigmentation of the retina reflects the green spectrum, enabling its filters to improve the retinal layer’s visibility and providing more data from underneath the retinal surface. Content about choroidal melanomas, choroidal nevi, choroidal ruptures, and pigmentary abnormalities is found in the red spectrum, which is exclusive to the choroidal layer under the pigmented epithelium [32,33].

A few of CFP’s drawbacks are its inconsistent pigmentation and Drusen look, its inability to resolve fundus depth, and its dearth of comprehensive quantitative data. The microscopic alterations inside the retina that are indicative of the disease’s early stages cannot be observed in FP. Therefore, OCT image interpretation may be used to acquire it [34]. In addition to the costly equipment, the spherical form of the globe results in image distortion, artifacts from eyelashes, and misleading conclusions from insufficient color representation. For fundus imaging, normal 30° fundus photography continues to be the best option. It takes a long time to acquire stereo images, and patients have to endure twice as many light flashes. To fuse the image stereoscopically and produce depth, image interpretation takes time and specific goggles or optical viewers. Large-scale clinical studies and ordinary clinics can still use CFP, despite these drawbacks and the introduction of newer imaging technologies [35].

### 2.2. Optical Coherence Tomography (OCT) Modality

**Figure 4:**
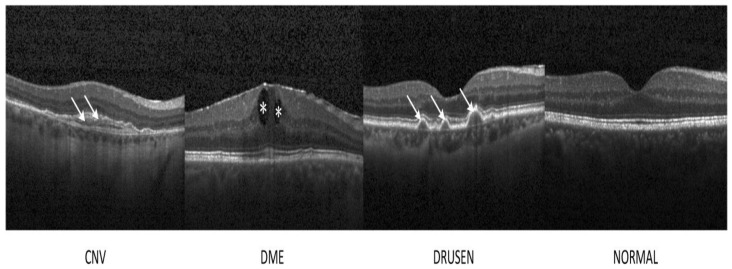
Representative OCT images. The lesion sites were denoted by asterisks and arrows. CNV: choroidal neovascularization; DME: diabetic macular edema, DRUSEN (AMD) and Normal [39].

OCT has developed into a potent imaging technique for the non-invasive evaluation of a variety of retinal disorders, including helping to diagnose DME, DRUSEN (AMD), and CNV, as shown in Figure 4. OCT is a high-resolution imaging, micron-level, and non-invasive method that provides real-time retinal imaging by utilizing infrared light with a wavelength range of 800–840 nm. The Michelson interferometry principle is the foundation of OCT. A light beam is split into two channels, one from the reference mirror and the other from the targeted tissue. The beams are then bounced back and combined again using semitransparent mirrors to create an interference pattern [36].

Since the method was first described in 1991, OCT imaging has quickly become commercially available, with advancements in technology enabling faster and higher-resolution imaging. The slower and more antiquated time domain OCT has mostly been superseded by Fourier- or spectral-domain OCT technology (SD-OCT). The technique’s main advantages include a much deeper tissue penetration, a much better ability to penetrate opaque media than SD-OCT, and a wider imaging depth that allows for high-quality imaging of the choroid and sclera at a collection rate that is at least four times faster (100,000 scans/s). Retinal thickness has been frequently measured by OCT for the assessment of ME resulting from conditions such as hereditary retinal degenerations, DR, AMD, ERM, cataract surgery, following RVO and uveitis [37]. OCT has several drawbacks. OCT uses light waves, hence medium opacities can impede the best possible imaging. Consequently, in the presence of vitreous hemorrhage, thick cataracts, or corneal opacities, the OCT will be restricted. The cost of OCT equipment is high. The quality of the image is influenced by the operator’s skill and may suffer when there is media opacity. Age, gender, and race-specific normative data are few, making it difficult to compare eyes with retinal illness [38].

### 2.3. Optical Coherence Tomography Angiography (OCTA) Modality

In recent years, OCT Angiography (OCTA) has been developed. It is an imaging method based on OCT that makes it possible to see the eye’s functioning blood vessels. This method is especially useful for the detection of microvascular alterations in the retina and choroid, which are indicative of illnesses that proceed slowly, including AMD, as was covered in [40,41,42,43,44]. The idea behind OCTA is to image blood flow by using the contrast mechanism created by moving particles, including red blood cells, which generate variations in the OCT signal. OCTA can identify erythrocyte mobility. It is quick, which gives it a significant benefit over other techniques for assessing patients who need to see you often.

**Figure 5:**
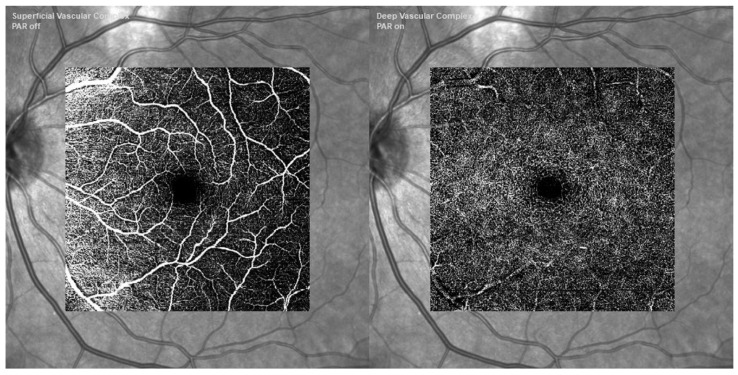
A patient with intermediate AMD was imaged using retinal imaging. Deep vascular complex OCTA (**bottom right**), superficial vascular complex OCTA (**bottom left**). Images captured with Heidelberg Engineering Ltd.’s Spectralis OCTA Module at Hemel Hempstead, UK [47].

Unlike the two-dimensional images of FA and ICGA, it is three-dimensional, making it possible to see the precise location and size of a lesion. Since foreign dyes are not needed for OCTA, it is non-invasive and safer than standard angiography. It enables direct in vivo visualization of the retinal and choroidal vasculature without staining or dye leakage, which might potentially hide the boundaries and morphology of diseases. Additionally, there is a distinction between the two primary OCTA technologies: spectral-domain (SD)-OCTA and swept-source (SS)-OCTA. The majority of commercial SD-OCTA devices employ a lower wavelength, about 840 nm, which is significantly attenuated by the RPE. Sub-RPE tissue attenuation may have a more significant role in Drusen or RPE thickening because the RPE functions as a significant hyperreflective barrier, obstructing light transmission. SS-OCTA employs a longer wavelength—roughly 1050 nm and can reach deeper into the choroid as presented in Figure 5 [45,46].

### 2.4. Fluorescein Angiography (FA) Modality

In the 1960s, Novotny and Alvis introduced fluorescein angiography (FA) to the field of ophthalmology. While FA is still highly helpful in certain situations, OCT is now more commonly employed to assess the response to therapy. One of the most important methods for identifying and categorizing CNV and its activity in eyes with neovascular AMD is FA. It is a diagnostic process that is intrusive and facilitates the evaluation of retinal and choroidal circulation pathology. It assists in the identification of several ocular illnesses, including AMD as discussed in [48,49].

It aids in the planning of decisions related to the management of ocular pathology. The formation of aberrant blood vessels under the retina’s RPE layer, known as CNV, is a defining feature of both dry and wet types of macular degeneration. These blood vessels have the potential to bleed, which may eventually lead to macular scarring and a significant loss of central vision (i.e., disciform scar). A specialized fundus camera with barrier and excitation filters is necessary for FA. Antecubital veins are typically used to provide fluorescein dye intravenously quickly enough to get high contrast early phase angiogram images. A blue excitation filter is applied to white light coming from a flash.

**Figure 6:**
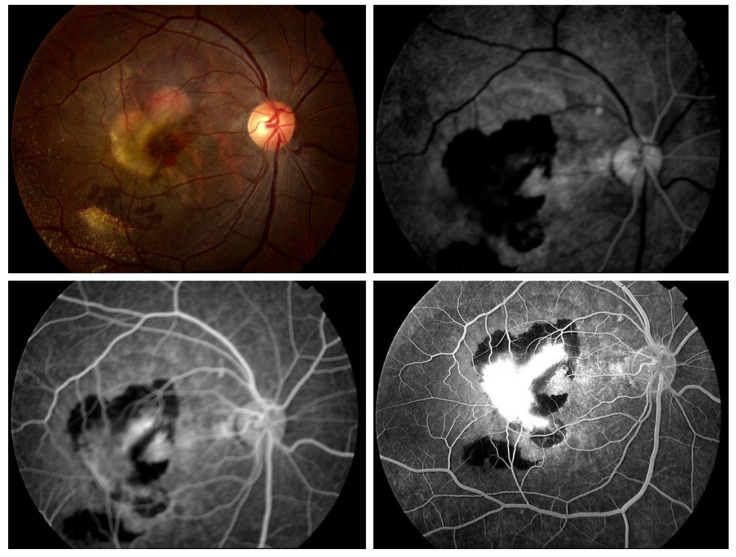
The FA imaging of the classic choroidal neovascular membrane in AMD shows early hyper fluorescence that increases in intensity over subsequent images, encircled by hypofluorescence from subretinal hemorrhage occlusion [51].

Following their absorption by free fluorescein molecules, blue light (wavelength 465–490 nm) is converted to green light (520–530 nm), which has a larger wavelength. To capture just the light released by the fluorescein in the photographs, a barrier filter eliminates any reflected light. After injection, images are taken right away, and they stay that way for fifteen minutes, depending on the pathology being examined. The images are taken either on 35mm film or digitally as shown in Figure 6 [50].

### 2.5. Indocyanine Green Angiography (ICG) Modality

Initially introduced in 1973 to investigate the choroidal circulation in ophthalmology, ICG angiography gained widespread usage in the early 1990s. For the identification of several retinal and choroidal disorders Including AMD as described in [52], ICG is a well-established method. With a molecular weight of 775, indocyanine green is a tricarbocyanine dye that is soluble in water. When it comes to the therapeutic usefulness of choroidal circulation visualization, two characteristics of ICG are significant. ICG first absorbs in serum between 790 and 805 nm, peaking at 835 nm. Secondly, ICG is a dye that is 98% attached to proteins and has a restricted diffusion via the choriocapillaris’s tiny fenestrations. The near-infrared spectrum is where both the excited and emitted light are found, which enables deeper penetration into the retina and easier passage of the emitted light through blood, lipid deposits, pigment, and moderate opacities (i.e., cataracts) to produce images.

**Figure 7:**
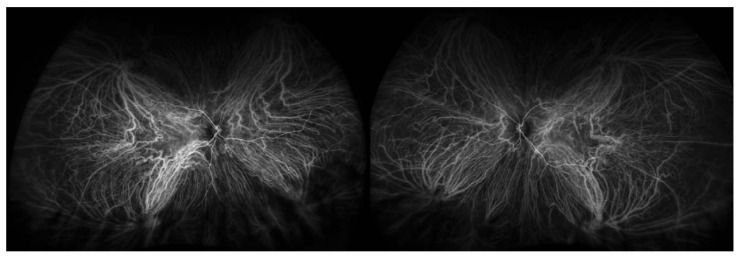
Bilateral wide field indocyanine green angiography [54].

Moreover, the dye typically stays inside the fenestrated walls of the choriocapillaris, as opposed to fluoresce, which leaks freely from these vessels, because it has a much higher molecular weight and a larger percentage of molecules stay bound to proteins in the bloodstream than fluorescein. Because of this characteristic, ICG is a perfect method for depicting the choroidal veins’ architecture and hemodynamics. High-definition images are produced by scanning laser ophthalmoscopes used for ICG angiography. ICG filters are also included in ultrawide field systems, which can produce ICG images with a 200-degree field of view as shown in Figure 7 [53,54].

Table 2 compares between the different imaging modalities: Color Fundus Photographs (CFP), Optical Coherence Tomography (OCT), Optical Coherence Tomography Angiography (OCTA), Fluorescein Angiography (FA), and Indocyanine Green Angiography (ICG).

## 3. AMD Diagnosing Framework

**Figure 8:**
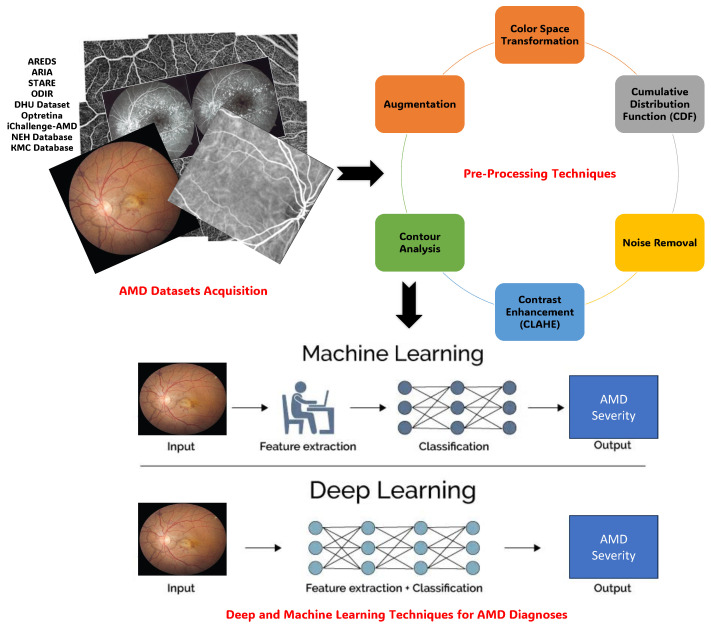
Graphical visualization of the AMD diagnosing framework that encompasses three main components: AMD Datasets Acquisition, Pre-Processing Techniques, and Deep and Machine Learning Techniques for AMD Diagnoses.

The AMD diagnosing Framework encompasses three main components: AMD Datasets Acquisition, Pre-Processing Techniques, and Deep and Machine Learning Techniques for AMD Segmentation and Diagnoses. The section on AMD Datasets Acquisition highlights both public and private databases crucial for research in AMD, such as STARE, DRIVE, and the AREDS database, each offering unique advantages and insights. Pre-processing techniques are then discussed, focusing on optimizing image quality, noise removal, contrast enhancement, and segmentation masking to prepare fundus images and OCT scans for analysis using ML/DL approaches. Lastly, Deep and Machine Learning Techniques for AMD Diagnoses utilize the rich landscape of methodologies harnessing AI, particularly DL, to enhance diagnostic capabilities. Techniques like CNNs, RNNs, and ensemble learning are explored, along with studies employing transfer learning and handcrafted features for classification. The performance evaluation metrics section concludes the framework, highlighting fundamental criteria such as accuracy, specificity, and sensitivity essential for assessing algorithm effectiveness and reliability in fundus image analysis. This comprehensive framework, as presented in Figure 8, provides a roadmap for advancing research and innovation in AMD diagnosis.

### 3.1. AMD Datasets Acquisition

This section provides a comprehensive overview of major public and private image databases integral to recent literature. The majority of public databases were produced with OCT and FP images [55]. The landscape of data repositories for advancing research in AMD is diverse, ranging from public resources such as DRIVE and STARE to proprietary datasets designed for specific investigations. They are summarized in Table 3.

#### 3.1.1. Public Databases

Research groups have been compelled to establish and make public their datasets due to the growing necessity of validating or training models. Due to their better fundus image resolutions, the open-access databases STARE and DRIVE are two of the most popular retinal databases [56].

AREDS Age-Related Eye Disease Study (AREDS): a 12-year investigation, AREDS was conducted. The AMD situations of numerous patients were monitored during this time in the study. Cases of GA, cases of Neovascular AMD, and control patients were added to the study. For the length of the trial, retinal images of each patient’s left and right sides were obtained. The AMD severity of the images was rated by several ocular doctors. During the study, several individuals who had previously experienced moderate AMD symptoms progressed to more severe AMD stages. This database’s test, validation, and training sets consist of 86,770, 21,867, and 12,019 images of color fundus photography (CFP), respectively [57].

ARIA (Automatic Retinal Image Analysis): There are 450 images in the JPEG format ARIA database. Three groups of photographs are identified: one with AMD, one with DR, and one with a healthy control group. It took two skilled ophthalmologists to annotate the images [58].

STARE (Structured Analysis of the Retina): Out of the 400 retinal images in STARE, 40 have manually segmented blood vessels and labeled veins/arteries. Experts have labeled each image. The PPM format was used to compress the image data. Also included are algorithms for the identification of the optic nerve head (ONH). Thirteen distinct abnormalities were linked to a total of 44 diseases that were identified [59].

ODIR Dataset Ocular Disease Intelligence Recognition (ODIR): The structured dataset known as ODIR consists of 5000 patients from the Peking University National Institute of Health Sciences. Eight retinal diseases (i.e., Diabetes, AMD, myopia, glaucoma, hypertension, cataract, normal, and others) are represented by the various label annotations in it. The JPEG format saves images in various sizes. The following is the distribution of the labels for the image classes: Typical: 3098, Glaucoma: 224 Diabetes: 1406, 265 is the cataract—242 Pathological Myopia, Hypertension: 107, AMD: 293, and Additional diseases: 791. The annotating exercise involved ophthalmologists with expertise [60].

DHU Dataset: 45 OCT collections from Duke University, Harvard University, and the University of Michigan are included in a publicly accessible dataset. This dataset includes 15 participants for each of the three classes (i.e., normal, DME, and dry AMD) consisting of volumetric scans using non-unique procedures [20].

Optretina Dataset: Since 2013, Optretina has conducted telemedicine screening programs in optical centers, and since 2017, it has expanded to include commercial businesses and workplaces. The 306,302 retinal images that make up the labeled dataset from Optretina serve as the basis for this investigation and are tagged with quality, laterality, and diagnostic. All of these images have been assessed by ophthalmologists, who are all specialists in retinal diseases; 20% of the data set has abnormal retinas, whereas the remaining 80% have normal ones. It is anonymized and based on real-world instances rather than clinical trials. Images are obtained using OCT and NMC (color fundus and red-free). The collection contains images taken with many brands and models of cameras. The collection contains additional non-medical images intended to trick the AI, as well as retinal nerve fiber layer OCTs (RNFL), macular OCTs, and CFP [61].

iChallenge-AMD: The AMD reference standard existence derived from the medical records takes into account Visual Field, OCT, and other information in addition to fundus images alone. The image folder contains AMD and non-AMD labels (also known as the reference standard) that are utilized for training data [62].

UMN: The University of Minnesota Ophthalmology Clinic collected 600 OCT B-scan images from 24 individuals who were diagnosed with exudative AMD, and this dataset is freely accessible. Every patient received about 100 B-scans, which is how the Spectrally technology took the images. Twenty-five of the images with the biggest fluid area were chosen to serve as the samples. Two ophthalmologists annotated and validated the retinal fluid, SRF, IRF, and PED. This database is utilized for the segmentation process [63].

OPTIMA: Through the cyst segmentation challenge at the Medical Image Computing and Computer-Assisted Intervention (MICCAI) 2015 conference, the OPTIMA dataset was made publicly available, and it has subsequently gained popularity as a resource for IRF Fluid/Cyst segmentation in OCT. Thirty volumes of patient data from different OCT equipment, such as Spectralis, Topcon, Cirrus, and Nidek, make up the dataset. Every retinal OCT volume is centered on the macula and is around 6 × 6 × 2 mm^3^. In order to better represent the wide variety of scans seen in clinical settings as well as the various appearances and distributions of IRF, the dataset has been further split into 15 training and 15 testing volumes. Two medical University of Vienna expert graders annotated the IRF in the training data. This database is utilized for the segmentation process [64].

RETOUCH: The RETOUCH dataset was obtained from the 2017 MICCAI conference’s retinal OCT fluid challenge, in which the OCT images were labeled with the following retinal fluid (fluid/cyst) labels: IRF, SRF, and PED. Seventy OCT volumes obtained from individuals diagnosed with macular edema related to AMD or RVO comprise the dataset. Twenty-four of these volumes came from the Spectralis OCT system, twenty-two from the Triton OCT system, and twenty-four from the Cirrus OCT system. Under the guidance of retina specialists from the Medical University of Vienna and Radboud University Medical Centre in the Netherlands, human graders annotated the images. There are 128, 128, and 49 B-scan images total that were obtained by the Cirrus, Triton, and Spectralis OCT systems, respectively. The resolution of each image varies. Much pertinent research on this subject has been based on the RETOUCH dataset, which is frequently used as a benchmark for assessing the effectiveness of retinal cyst/fluid segmentation algorithms [65].

VRC: The Vienna Reading Centre offers top-notch imaging services for ophthalmology-related clinical, pharmacological, and scholarly research. Established in 2005 as the world’s first entirely digital image analysis platform, the VRC is a division of the Medical University of Vienna’s Department of Ophthalmology, which is among the biggest medical schools in Europe. Their group’s main area of interest is the creation of computational image analysis techniques. Close cooperation with the Department of Ophthalmology’s independent research unit, the Christian Doppler Laboratory for Ophthalmic Image Analysis (OPTIMA), fosters this effort comprising 1200 full OCT volume scans of the eyes of patients afflicted with AMD, DME, and RVO, the three main disorders known to cause macular fluid. This database is utilized for the segmentation process [66].

#### 3.1.2. Private Databases

Using private databases, researchers have also evaluated algorithm performance in the AMD detection area. Images are anonymized in compliance with ethical standards and subject to privacy protection before being used for model design and performance assessment. In certain instances, the medical facilities and authors that own the data or funded the study may be able to access certain private databases upon request.

NEH Database: The Noor Eye Hospital in Tehran is the source of the NEH dataset, which is made up of 48 dry AMD, 50 DME, and 50 normal OCTs. The axial resolution in this dataset is 3.5 μm, and the scan dimension is 8.9 × 7.4 mm^2^. However, not all patients have the same lateral and azimuthal resolutions. Consequently, the number of A-scans varies from 512 to 768 scans, while different patients provide 19, 25, 31, and 61 B-scans per volume. The dataset is available for access at http://www.biosigdata.com (accessed on 8 July 2024) [20].

KMC Database: Ophthalmology Department of Kasturba Medical College (KMC), Manipal, India from Kasturba Medical Hospital. There are 402 eyes in the fundus image dataset that are normal, 125 retinal images that show wet AMD, and 583 early, intermediate, or GA AMD retinal images. Featuring a pixel resolution of 2588×1958, the Zeiss FF450 plus mydriatic fundus camera was used to capture the images [1].

KSH Database: The database consists of 775 high-resolution color fundus images from patients with early AMD from the Department of Ophthalmology at Kangbuk Samsung Hospital, taken between 2007 and 2018, are included in the collection. Nonmydriatic fundus cameras from many manufacturers were used to capture fundus images: Topcon, Japan made the TRC NW300, TRC-510X, NW200, and NW8, Canon, Japan made the CR6-45NM and CR-415NM, and Zeiss, USA made the VISUCAM 224. The fundus images’ digital images were examined using an image archiving communication system (INFINITT, Seoul, Republic of Korea) [67].

An overview of the retinal image databases that were previously addressed can be found in Table 3, which also includes information on the number of images, resolutions, cameras, and purposes for which the databases were made.

**Table 3:** Tabular summary of public and private available AMD datasets.

Dataset	Description	Images	Purpose	Resolution	Annotation	Availability	Modality
AREDS [57]	Longitudinal study monitoring AMD progression	86,770	Classification	Various	Graded by ocular experts	Public	CFP
ARIA [58]	450 JPEG images with AMD, DR, and healthy controls	450	Classification	Various	Annotated by ophthalmologists	Public	CFP
STARE [59]	400 retinal images with manually segmented blood vessels	400	Classification, Segmentation	(700×605)	Expert-labelled abnormalities	Public	CFP
ODIR [60]	Contains 8 retinal diseases (i.e., Diabetes, AMD, myopia, glaucoma, hypertension, cataract, normal, and others) from the Peking University National Institute of Health Sciences	5000	Classification	Various	Annotations by ophthalmologists	Public	CFP
DHU Dataset [20]	45 OCT volumes with normal, DME, and dry AMD from Duke University, Harvard University, and the University of Michigan	45	Classification	Various	Expert-labelled classes	Public	OCT
Optretina [61]	labeled retinal images from telemedicine screening programs.	306,302	Classification	Various	Annotated by ophthalmologists	Public	CFP, OCT
iChallenge-AMD [62]	Fundus image dataset with AMD and non-AMD labels	-	Classification	Various	Reference standard derived from medical records	Public	CFP, OCT
UMN Database [63]	600 OCT B-scan images from the University of Minnesota Ophthalmology Clinic	600	Segmentation	Various	Ophthalmologists	Public	OCT
OPTIMA Database [64]	Cyst segmentation challenge at the MICCAI 2015 conference	15 training, 15 testing volumes	Segmentation	Various	Medical University of Vienna experts	Public	OCT
RETOUCH Database [65]	Retinal fluid (fluid/cyst) labels: IRF, SRF, and PED from the 2017 MICCAI conference’s retinal OCT fluid challenge	70 volumes	Segmentation	Various	Retina specialists	Public	OCT
VRC Database [65]	Eyes of patients afflicted with AMD, DME, and RVO, three disorders known to cause macular fluid from Medical University of Vienna’s Department of Ophthalmology	1200 volume	Segmentation	Various	Retina specialists	Public	OCT
NEH Database [20]	OCT dataset from Noor Eye Hospital, Tehran	148	Classification	3.5 μm axial resolution	Anonymized annotations	Private	OCT
KMC Database [1]	Fundus image dataset from Kasturba Medical College	1110	Classification	2588×1958	Expert annotations	Private	CFP
KSH Database [67]	High-resolution images from patients with early AMD from Department of Ophthalmology at Kangbuk Samsung Hospital	775	Classification	Various	Expert annotations	Private	CFP

### 3.2. Pre-Processing Techniques

Pre-processing of fundus images and OCT scans is crucial for optimizing image quality, removing noise, and preparing them for analysis with ML/DL approaches. This step ensures the development of robust prediction models for challenges such as uneven illumination, noise, artifacts, and other imperfections that could affect the analysis process [31,68].

Various techniques enhance DL model performance in analyzing fundus images and OCT scans. Color Space Transformation selectively uses a single color channel, often eliminating the green channel to avoid interference with analysis, especially in high-contrast images. The Cumulative Distribution Function (CDF) helps understand pixel intensity distribution, providing insights into image characteristics [69].

Noise Removal methods, such as non-local means denoising and median filters, ensure cleaner and more accurate analysis results. Contrast Enhancement, particularly through methods like Contrast Limited Adaptive Histogram Equalization (CLAHE), improves the visibility of small lesions like microaneurysms. Segmentation Masking isolates regions of interest (ROIs) within fundus images, enhancing diagnostic accuracy by focusing on specific areas for analysis. Contour Analysis refines ROIs and identifies object boundaries, aiding in modifying ROIs and determining object attributes [70].

Augmentation techniques, such as rotation and flipping, balance image datasets, ensuring robustness in DL models. Cropping and Extracting Regions of Interest (ROI) isolate areas containing significant information, reducing unnecessary learning effort [71]. Histogram Equalization enhances contrast, improving image clarity, while resizing images to standard dimensions maintains consistency across the dataset. Lastly, Enhancing Image Contrast reduces noise and enhances visual clarity for more accurate analysis [72]. Table 4 summarizes these frequently employed pre-processing techniques for fundus images utilized in diagnosing retinal diseases.

### 3.3. Deep and Machine Learning Techniques for AMD Segmentation

Image segmentation is necessary for a quantitative assessment of the lesion area [73]. Researchers have been concentrating more and more on using DL and standard ML techniques to automate the segmentation of retinal cysts and fluid in recent years. A fluid-filled pocket in the retina known as a retinal cyst or fluid is a pathological result of several common eye conditions, such as AMD. Ophthalmologists can benefit greatly from the automated procedures by using them to better interpret and quantify retinal characteristics, which can lead to more precise diagnosis and well-informed treatment options for retinal illnesses. AMD may be diagnosed with the aid of Drusen, the primary disease manifestation. The aberrant deposition of metabolites from RPE cells is the cause of them. The process of Drusen segmentation faces four primary obstacles: Their color is comparable to that of the fundus image and OD, with a yellowish-white hue; they frequently have irregular forms and indistinct borders, as well as uneven brightness and interference from other indicators like blood vessels. Several Drusen segmentation technologies are available in the field of image study in ophthalmology.

Yan et al. [74] suggested a deep random walk technique for effectively Drusen segmentation from fundus images. There are three primary components to the suggested architecture. First, fundus images are sent into a deep feature extraction module, which is divided into two branches: a low-level feature-capturing three-layer CNN and a deep semantic feature-capturing SegNet-like network. Subsequently, the acquired data are combined and sent into a designated affinity learning module to derive pixel-by-pixel affinities for creating the random walk’s transition matrix. Lastly, manual labels are propagated using a deep random walk module. On the STARE and DRIVE datasets, this model demonstrated state-of-the-art performance with accuracies of 97.13, sensitivity of 92.02, and specificity of 97.30. The acquired deep characteristics can assist in managing Drusen variations in size and form as well as color resemblance to different tissues. Furthermore, accurate segmentation is achieved at hazy Drusen borders via the random walk approach.

Contributions: A deep random walk method for Drusen segmentation from fundus pictures is presented in this research. The technique’s strength lies in the random walk process driving the learning algorithms for pixel-pixel affinities and deep picture representations. Compared to other cutting-edge methods, their network is more capable of handling the difficulties associated with Drusen segmentation because of its ability to handle Drusen fluctuations in size and form as well as color resemblance to other tissues thanks to its learned deep characteristics. Furthermore, accurate segmentation is achieved at hazy Drusen borders via the random walk approach.

Another work was proposed, based on Drusen segmentation for AMD detection, by Pham et al. [67] since there were far more non-Drusen pixels than Drusen pixels, they attempted to address the issue of data imbalance by employing a multi-scale deep learning model. Other studies attempted to analyze cropped images, which may lose global information, in an attempt to tackle the high-resolution image problem. Other than cropping the central image to eliminate unnecessary background areas, they do not use any particular pre-processing procedures. The associated approach consists of two networks: a patch-level network that makes final predictions based on related patch images and associated probability maps, and an image-level network that generates Drusen probability maps using a Deeplabv3+ base architecture. The U-Net-based segmentation is used by the Patch level network. For training, a total of 775 fundus photographs from the Samsung Hospital in Kangbuk were utilized. The STARE dataset was used to assess the model’s performance as well. Using data from Kangbuk Samsung Hospital, this model produced an accuracy of 0.995, sensitivity of 0.662, specificity of 0.997, and dice score of 0.625. Using the STARE dataset, this model produced results with an accuracy of 0.981, sensitivity of 0.588, specificity of 0.991, and dice score of 0.542.

- Contributions: Their approach predicts more accurate Drusen segmentation masks by integrating local and global data. Furthermore, they can enhance the accuracy of Drusen detection in the early stages of AMD by utilizing the pre-trained model and a mix of several loss functions. They attempted to address the issue of data imbalance by employing a multi-scale deep learning model.
- Limitations: There are many restrictions on the model. Binary segmentation may be done with the present configuration. In multi-class segmentation, the Patch-Level Network’s input channel count grows in proportion to the number of classes. It may result in issues with computational complexity and scalability. Furthermore, their approach has to forecast many patches for every high-resolution fundus image to get the Drusen prediction. As a result, their model may require a longer inference time than the others.

Several CNN and FCN designs have been put out since 2017 to segment the cyst/fluid area in OCT images. Assigning a label to every pixel in an image is the goal of semantic segmentation tasks, which are especially suited for FCNs, a subset of CNNs. FCNs have a structure akin to CNNs, but they use a distinct design that preserves the image’s spatial information across the network. Using CNNs for image identification tasks has several benefits, including the ability to handle high-dimensional data, like images, automatically learn and extract features from data, and generalize well to new data. In addition to using CNN’s advantages, FCN can handle complex prediction jobs like semantic segmentation.

Chen et al. [75] suggested a quicker R-CNN-based deep learning-based segmentation method to segment various retinal fluids, such as the pigment epithelium detachment (PED) areas, sub-retinal fluid (SRF), and intra-retinal fluid (IRF). To prevent overfitting, they first segmented the IRF areas and then enlarged the regions to segment the SFT. The PED areas were then divided using the RPE layer. Three distinct forms of retinal SD-OCT volumes from various equipment, Cirrus, Spectralis, and Topcon, are used for the research. Using a general approach to separate different types of illnesses was challenging. As a result, they created various segmentation strategies for various illnesses. The segmentation of IRF was done using a quicker R-CNN-based approach, the segmentation of SRF was done using a 3D region growing-based method, and the segmentation of PED was conducted using an RPE layer segmentation-based method. Three approaches provide passable outcomes. For lesion locations, this approach almost attains an overlapping ratio of 60%.

- Contributions: This research presented a quicker R-CNN-based deep learning-based segmentation method to segment various retinal fluids. They created various segmentation strategies for various illnesses. Three approaches provide passable outcomes.
- Limitations: The highest overlapping ratio for three types of lesion areas is close to 0.7. The reason is that it is hard to detect lesion areas whose boundary is not obvious and the area is very small. Also, SRF and PEDs interfere with each other seriously.

On the other hand, Schlegl et al. [66] developed an eight-layer FCN for end-to-end segmentation using a dataset made up of 1200 OCT scans from AMD, DME, or RVO patients. A deep learning-based technique was created to automatically identify and measure subretinal fluid (SRF) and intraretinal cystoid fluid (IRC). The Medical University of Vienna’s Ethics Committee acquired the dataset. With a (AUC) of 0.94, a mean precision of 0.91, and a mean recall of 0.84, they were able to obtain excellent accuracy for the identification and measurement of IRC for all three retinal diseases. With an AUC of 0.92, a mean precision of 0.61, and a mean recall of 0.81, the identification and measurement of SRF were likewise quite accurate. This was especially true for neovascular AMD and RVO, which performed better than DME, which was not frequently seen in the population under study. Between automated and manual fluid localization and quantification, a high linear correlation was verified, resulting in an average Pearson’s correlation coefficient of 0.90 for IRC and 0.96 for SRF.

- Contributions: They created and validated a completely automated technique to identify, classify, and measure macular fluid in standard OCT scans. For all three retinal diseases, the newly developed, totally automated diagnostic approach based on deep learning obtained excellent accuracy for IRC identification and quantification. Moreover, fluid quantification obtains a high degree of agreement with expert manual evaluation.
- Limitations: There is room for improvement based on the SRF detection performance in DME eyes. The decreased performance can be attributed to the fact that DME eyes often have a lower prevalence and less SRF than neovascular AMD or RVO. As a result, the model is unable to adjust for the various class label distributions when the approach is trained only on AMD and RVO instances and then applied to DME situations. However, because of the limited number of target labels, the approach would not be able to obtain a satisfactory representation for SRF when it was directly trained on DME examples. Simultaneous training on AMD, RVO, and DME instances would result in reduced test performance on DME because of changes in label distributions. A significant number of ground truth situations should be available to get around this restriction.

Also, ASPP contributed to Sappa’s work on RetFluidNet, Sappa et al. [76] demonstrated the use of RetFluidNet, an enhanced convolutional neural network (CNN)-based architecture, to separate three different kinds of fluid abnormalities from SD-OCT images. The RetFluidNet design could segment three types of retinal fluids: IRF, SRF, and PED. The RetFluidNet did that by integrating skip-connect techniques with ASPP, the author additionally looked at the effects of hyperparameters and skip-connect approaches on fluid segmentation. B-scans from 124 patients were used for training and testing RetFluidNet and all the comparison techniques. A Cirrus SD-OCT machine (Carl Zeiss Meditec, Inc., Dublin, CA) provided the B-sans. For IRF, PED, and SRF, RetFluidNet obtained accuracy values of 80.05%, 92.74%, and 95.53%, correspondingly. When compared to competing efforts, RetFluidNet showed a notable improvement in clinical applicability, respectable accuracy, and time efficiency.

- Contributions: A completely automated technique called RetFluidNet helps to separate three different kinds of fluid abnormalities from SD-OCT images and this leads to AMD early identification and follow-up.
- Limitations: They did not investigate the impact of downsampling on the tiny fluid areas. Given that there could be tiny fluid pockets, particularly for IRF, it might be wise to look at the effects of downsampling in the future and develop a different method for scaling images. The primary obstacle faced by the work was the complex optimization procedure that resulted from the numerous hyperparameters and their interdependencies. It takes time to examine the impact of changing the hyperparameters, which makes hyperparameter tuning laborious and thorough.

Kang et al. [77] designed a two-phase neural network for fluid-filled areas segmentation such as pigment epithelial detachment (PED), subretinal fluid (SRF), and intraretinal fluid (IRF) in retinal OCT images. Using the RETOUCH challenge, a satellite event of the MICCAI 2017 conference. The first network’s objective was to identify and divide fluid areas, while the second network enhanced the first one’s resilience. To prevent overfitting during training, the network used a U-net design with a classification layer and max-out and dropout activation. The loss function of the neural network was altered for the U-Net design.

Contributions: In this study, a deep learning approach for fluid segmentation and image identification from OCT images was developed. They created the two-step network to increase the network’s resilience. The suggested approach performs similarly for every data set, even though the three suppliers’ images have distinct qualities.

To enhance the segmentation performance, changes to the loss functions have also been taken into consideration in addition to changes to the network topologies. Liu et al. [78] prevented nearby areas from being recognized as a single entity and addressed the problem of tiny fluid area detection using an uncertainty-aware semi-supervised framework. The RETOUCH dataset was used to assess this approach. The framework was made up of two networks: one for teachers and the other for students. Both networks used the same architecture and three decoders to forecast the probability, contour, and distance maps of the image, which allowed for segmentation. The teacher network could direct students toward the acquisition of more trustworthy information by restricting consistency loss.

- Contributions: Suggested a semi-supervised, uncertainty-aware architecture for segmenting retinal fluid. These networks have decoders that can predict the probability, contour, and distance maps at the same time. Their approach can enhance the network’s prediction power on the fluid’s boundary while reducing the instability of the nearby fluid regions that are merging.
- Limitations: Some IRF areas are merged by ALNet. ASDNet can segment certain tiny fluid areas and separate some near-fluid regions in comparison to ALNet. Certain false positive areas can be rejected by RFCUNet. Some fluid zones did, however, continue to combine.

Deep learning research in segmentation utilizes the U-Net architecture, a popular type of Fully Convolutional Network (FCN) widely applied in medical image segmentation. The U-Net’s design features a contracting path and an expanding path, enabling precise feature localization and effective network training. This architecture has proven effective in various biomarker segmentation studies. For instance, Tennakoon et al. [79] introduced a method based on U-Net architecture trained with a mixed loss function incorporating dice, adversarial loss terms, and cross-entropy. The inclusion of adversarial loss enhances the encoding of higher-order pixel correlations, eliminating the need for separate post-processing in segmentation tasks. Their approach focused on voxel-level segmentation of three types of fluid lesions (IRF, SRF, and PED) in OCT images from the ReTOUCH challenge, demonstrating the efficacy of deep neural networks with integrated adversarial learning for accurate medical image segmentation.

Contributions: They suggested a deep learning-based approach to the segmentation and categorization of retinal fluid in OCT images. The suggested approach is predicated on a modified U-Net architecture that was trained with a mixed loss function that included an adversarial loss term. The suggested technique has demonstrated efficacy in predicting the existence and voxel-level segmentation of retinal fluids, according to validation data. Retinal fluid segmentation has improved as a result of the network design and loss functions employed.

Diao et al. [80] developed a complementary mask-guided CNN (CM-CNN) as part of a deep learning framework to perform the classification of OCT B-scans with Drusen or CNV from normal ones. The auxiliary segmentation task generated the guiding mask for the CNN. Second, utilizing the CAM output from the CM-CNN, a class activation map guided UNet (CAM-UNet) was presented to achieve the segmentation of Drusen and CNV lesions. The suggested dual guidance network outperformed five classification networks, four segmentation networks, and three multi-task networks in terms of both classification and segmentation accuracy when tested on a portion of the public UCSD dataset. The segmentation Dice coefficient was 77.51%, and the classification accuracy was 96.93%. The suggested model’s generalizability was further demonstrated by the results obtained by segmenting retinal fluids and detecting macular edema on an additional dataset.

Contributions: To classify OCT pictures, a complementary mask-guided CNN is suggested. An auxiliary job of lesion segmentation is added, and the segmentation mask that is produced is employed in a complementary form to guide the extraction of classification features. To accomplish autonomous AMD lesion segmentation, a class activation map guided UNet is suggested. The segmentation job is directed by the CAM from classification, which is fused into the features at each layer.

Table 5 summarizes the discussed machine and deep learning segmentation approaches.

### 3.4. Deep and Machine Learning Techniques for AMD Diagnoses

Exploring DL/ML Techniques for AMD diagnoses reveals a landscape rich in methodologies harnessing AI to enhance diagnostic capabilities. These techniques, particularly DL, analyze vast datasets with complex algorithms and neural networks, unveiling patterns beyond human capacity and automating AMD detection and classification. CNNs, inspired by human visual processing [81], excel in recognizing subtle features in retinal images, aiding early and accurate diagnoses of AMD [82,83]. Recurrent neural networks (RNNs) and Long Short-Term Memory Networks (LSTMs) analyze sequential data like OCT scans over time, crucial for tracking AMD progression [84].

Ensemble learning combines predictions from multiple models, enhancing diagnostic accuracy and generalizability across datasets. Transfer learning utilizes pre-trained models on large datasets to optimize efficiency, especially when annotated data are limited in medical imaging. This amalgamation of DL/ML techniques propels AMD diagnostics into a new era of precision. From CNNs to ensemble learning, these methodologies collectively redefine AMD assessment, promising a more efficient and accurate diagnostic landscape [85,86].

#### 3.4.1. Deep Learning Techniques

Recent advancements in DL for AMD classification are discussed in this section, showing promising results in categorizing retinal fundus images for different AMD stages. Studies have explored network depth and transfer learning, indicating that training networks from scratch with sufficient data can lead to higher accuracy than using pre-trained models [87,88]. However, addressing model limitations, such as generalizability to diverse datasets and matching attention maps with underlying vision transformer (ViT0 sickness, requires further research. In diagnosing retinal diseases, DL tasks mainly involve segmentation and classification. Classification directly categorizes input images into illness groups, while segmentation tasks reveal detailed information about retinal disorders from fundus images, including critical lesions and biomarkers [7,89,90,91].

Several studies have examined categorizing AMD modalities, using different techniques and methodologies. For example, Karri et al. [19] proposed a transfer learning method based on the Inception network, utilizing the DHU database to classify dry AMD, DME, or normal using a pre-trained CNN and GoogLeNet. Initially, saturated pixels were replaced with pixels of intensity 10. Following RPE estimation, smoothing, and retinal flattening, the RPE lower contour was relocated to a fixed position 71% of the height). The image was resized to 224×224 and filtered using the BM3D filter. Three BM3D filter results were replicated, each treated as channel information. The proposed method utilized flattened and filtered images to classify retinal OCT images as normal, DME, or AMD. Across all validations (N1, N2, and N3), the average prediction accuracy for normal, DME, and AMD was 99%, 86%, and 89%, respectively, with the best model achieving 94% accuracy.

Philippe et al. [92] utilized DL techniques, including transfer learning and universal features trained on fundus images, along with input from a clinical grader, to develop automated methods for AMD detection from fundus images. Their approach aimed to classify images into disease-free/early stages and referable intermediate/advanced stages using the AREDS database. The deep CNN achieved an accuracy ranging from 88.4% to 91.6% and an AUC between 0.94 and 0.96.

Li et al. [21] utilized the VGG16 to classify CNV and normal AMD using a private retinal OCT image database. They employed image normalization but did not incorporate image denoising to avoid overfitting and enhance generalization. To address variations in image intensities, they normalized and resized OCT volumes to ensure uniform dimensions for processing with VGG16. They developed a normalization technique to adjust eye curvature, normalize volume intensities, and align BM layers achieving 100% AUC, 98.6% accuracy, 97.8% sensitivity, and 99.4% specificity.

Bulut et al. [93] proposed a DL method for identifying retinal defects using color fundus images. They employed the Xception model and transfer learning technique, training on images from the eye diseases department of Akdeniz University Hospital, and additional open-access fundus datasets for testing. The study explored 50 potential parameter combinations of the Xception model to optimize performance. The fourth model achieved the highest accuracy of 91.39% for the training set, while the zeroth model attained the best accuracy of 82.5% for the validation set.

Xu et al. [94] devised a DL approach using a ResNet50 model on a private database from Peking Union Medical College Hospital. They also constructed alternative machine-learning models with random forest classifiers for comparison. Three retinal specialists independently diagnosed the same testing dataset for a human-machine comparison, with participant photographs presented in a single partition subset. Utilizing fundus and OCT image pairs, their dual deep CNN model classified polypoidal choroidal vasculopathy (PCV) and AMD. Transfer learning involved initially applying ResNet-50 weights to two separate models handling fundus and OCT images, refining weights on new data, and then transferring them to corresponding convolutional blocks. The final FC layer classified input pairs into four categories: PCV, Wet AMD, Dry AMD, and AMD. The bimodal DCNN outperformed the top expert (Human1, Cohen’s κ 0.810) with 87.4% accuracy, 88.8% sensitivity, 95.6% specificity, and full agreement with the diagnostic gold standard (Cohen’s κ 0.828).

- Contributions: Studies [19,21,92,93,94] showed the effectiveness of fine-tuning models trained on medical/non-medical images for improved disease recognition compared to traditional methods. It also highlighted the feasibility of utilizing pre-trained models for faster convergence with less data. Some studies compared their method with alternative machine-learning models, such as random forest classifiers.
- Limitations: Studies [19,21,92,93,94] encountered challenges such as overfitting, reliance on a private database, gradient disappearance or explosion and slight class imbalance in the number of fundus images during training were encountered. Additionally, some studies relied only on accuracy. Furthermore, some studies relied solely on the AREDS dataset for evaluation, lacking validation on independent clinical datasets such as MESSIDOR. Additionally, the reliance on transfer learning and a single model architecture may limit the generalizability of the approach to different datasets or clinical settings, suggesting the need for further validation and exploration of alternative methodologies.

Rasti et al. [20] developed a CAD system using public OCT images (NEH dataset; DHU dataset) to differentiate between dry AMD, DME, and normal retina. Their approach utilized a multi-scale convolutional mixture of expert (MCME) ensemble model, a data-driven neural structure employing a cost function for discriminative and fast learning of image attributes. Image preparation involved normalization, retinal flattening, cropping, ROI selection, VOI creation, and augmentation. On the DHU dataset, the model achieved accuracy, recall, and AUC of 98.33%, 97.78%, and 99.90% for N1, N2, and N3, respectively, while on the NEH dataset, it achieved total precision, total recall, and total AUC of 99.39%, 99.36%, and 0.998.

- Contributions: The study introduced a CAD system employing the MCME ensemble model for classifying OCT images into dry AMD, DME, or normal retina categories. By incorporating a mixture model, the study achieved high accuracy, recall, and AUC, demonstrating the effectiveness of CNNs on multiple-scale sub-images.
- Limitations: The study lacked occlusion testing and qualitative assessment of model predictions. Additionally, it did not include image denoising, complete retinal layer segmentation, or lesion detection algorithms, which are essential for comprehensive analysis in clinical settings.

Thakoor et al. [41] developed a custom-built 3D CNN with two dense layers, four 3D convolutional layers, and a final SoftMax classification. They discuss 3D–2D hybrid CNNs. Patients who were 18 years of age or older who were treated by collaborating vitreoretinal faculty at Columbia University Medical Centre provided the data utilized in this study. Patients with non-neovascular AMD, patients with no significant vascular pathology on OCTA (non-AMD), and patients with neovascular AMD who have actionable CNV based on patient diagnosis make up the three patient groups for CNN training. They achieve a 93.4% testing accuracy in the binary categorization of neovascular AMD vs. non-AMD by using stacked 2D OCTA images of the retinal layers with 97 healthy, 80 neovascular AMD (NV-AMD) and 160 non-neovascular AMD (non-NV-AMD).

- Contributions: The findings demonstrate the superiority of models with various imaging modalities concatenated into one, such as OCT volumes with b-scan images and OCTA, over models with a single input image modality. By using dataset balance, the models’ performance was improved. GradCAM visualization at each layer of the 3D input volumes indicates that model performance may be enhanced by including a high-resolution b-scan cube across the retina. Very modest angiographic results, particularly in early types of AMD, need to be confirmed by high-resolution b-scans.
- Limitations: Imbalanced settings and data-limited are the study’s limitations.

Tan et al. [1] proposed a deep CNN for diagnosing AMD using fundus images. Their custom-designed CNN, consisting of 14 layers, aimed to classify images into AMD (dry and wet) and normal categories. Utilizing data from the Ophthalmology Department of Kasturba Medical College (KMC), they employed blindfold and 10-fold cross-validation methods, achieving high classifier accuracies of 91.17% and 95.45%, respectively.

- Contributions: The study proposed a deep CNN for diagnosing AMD using fundus images, consisting of 14 layers, and aimed at classifying images into AMD (dry and wet) and normal categories. The model’s portability and affordability make it suitable for deployment in regions with limited access to ophthalmology services, facilitating rapid screening for AMD among the elderly.
- Limitations: The study faced limitations such as the need for large amounts of labeled ground truth data for optimal model performance. Additionally, the complexity of the CNN model led to issues with overfitting and convergence, requiring continuous parameter adjustments for optimal performance. Moreover, CNN model training was laborious and time-consuming, although testing of fundus images became quick and precise once the model was trained.

Jain et al. [95] aimed to classify retinal fundus images as diseased or healthy without explicit segmentation or feature extraction. They developed the LCDNet system for binary categorization using DL techniques. Two datasets were utilized: one from the ML data repository at Friedrich-Alexander University and another from the Retinal Institute of Karnataka, India. The model achieved an accuracy ranging from 96.5% to 99.7%. Notably, color images performed worse than red-free images, aligning with medical community beliefs.

- Contributions: The study developed the LCDNet system for binary categorization of retinal fundus images as diseased or healthy, utilizing DL techniques without explicit segmentation or feature extraction. The study highlighted the potential of DL for automated disease classification in retinal images, with red-free images showing better performance compared to color images, aligning with medical community beliefs.
- Limitations: The study identified the need for a larger and more diverse dataset to enhance the model’s performance for specific diseases. Additionally, the focus on binary categorization limits the model’s ability to distinguish between different diseases or severity levels within diseased images. Comprehensive training with multi-class datasets would be necessary to address this limitation and improve the model’s applicability in clinical settings.

Islam et al. [96] proposed a CNN-based method to identify eight ocular disorders and their affected areas. After standard pre-processing, data are fed into the network for classification. Using the ODIR dataset and a state-of-the-art GPU, the model achieved an AUC of 80.5%, a κ score of 31%, and an F-score close to 85%.

- Contributions: This study marked the first evaluation of various eye disorders on a real-life dataset, demonstrating strong performance across different datasets. Additionally, the model showed flawless performance when tested on alternative datasets, highlighting its accuracy in identifying all eight categories of ocular disorders. The user-friendly nature of the system and its practical viability in real-time testing underscore its potential for revolutionizing public healthcare services through image processing and neural networks.
- Limitations: The study did not address specific challenges or limitations encountered during model development or evaluation. Further exploration of potential limitations, such as computational resource requirements, dataset biases, or generalizability to diverse populations, could provide valuable insights for future research and implementation in clinical settings.

Bhuiyan et al. [97] utilized CNNs and the AREDS dataset to classify Referable AMD of fundus images into no, early, intermediate, or advanced AMD and predict late AMD progression: dry or wet. They developed six automated AMD prediction algorithms based on color fundus images by integrating DL, ML, and algorithms for AMD-specific image parameters. These methods produced image probabilities, which were combined with demographic and AMD-specific parameters in a machine-learning prediction model to identify individuals at risk of progressing from intermediate to late AMD.

- Contributions: The study utilized CNNs and the AREDS dataset to develop six automated AMD prediction algorithms, classifying Referable AMD of fundus images into various stages and predicting late AMD progression. By integrating DL, ML, and algorithms for AMD-specific image parameters, the study produced image probabilities combined with demographic and AMD-specific parameters, facilitating the identification of individuals at risk of progressing from intermediate to late AMD. The immediate applicability of these methods in AMD studies using color photography could significantly reduce human labor in image classification, potentially advancing telemedicine-based automated screening for AMD and improving patient care in the field of public health.
- Limitations: The study faced limitations, particularly in lower prediction accuracy when stratified according to GA and CNV. This discrepancy might be attributed to the predominance of non-incident events compared to pure dry and wet AMD cases during the construction of machine-learning models.

Zapata et al. [61] utilized the Optretina dataset, comprising approximately 306,302 images, to develop a CNN-based classification algorithm for AMD Disease vs. No disease. They designed five algorithms and involved three retinal specialists to classify all images. Three CNN architectures were employed, two of which aimed to reduce parameters while maintaining accuracy. The study achieved an accuracy of 0.863, an AUC of approximately 0.936, a sensitivity of 90.2%, and a specificity of 82.5%.

- Contributions: The study utilized the Optretina dataset to develop a CNN-based classification algorithm for AMD Disease vs. No disease, involving three retinal specialists in image classification. The study designed five algorithms and employed three CNN architectures, achieving impressive performance metrics. These algorithms demonstrated the ability to assess image quality, distinguish between left and right eyes, and accurately identify AMD and GON with notable sensitivity, specificity, and accuracy.
- Limitations: The study may have faced limitations such as potential biases introduced by the involvement of retinal specialists in image classification. Additionally, the performance metrics achieved might vary across different datasets or clinical settings, warranting further validation and evaluation on diverse datasets to ensure the generalizability and robustness of the algorithms.

Yellapragada et al. [98] developed a deep neural network with self-supervised Non-Parametric Instance Discrimination (NPID) to predict AMD severity in four steps (none, early, intermediate, and advanced AMD) and classify the disease as referable AMD (intermediate or advanced AMD) and advanced AMD (CNV or central GA). They utilized the Age-Related Eye Disease Study (AREDS) and applied three-step, four-step, and nine-step classification methods to evaluate the model’s performance in grading AMD severity without labels. Using spherical k-means clustering and hierarchical learning, they studied network behavior and compared balanced and unbalanced NPID accuracies against ophthalmologists and supervised-trained networks. NPID demonstrated flexibility in different AMD classification schemes and achieved balanced accuracies comparable to human ophthalmologists or supervised-trained networks in classifying advanced AMD (82% vs. 81–92% or 89%), referable AMD (87% vs. 90–92% or 96%), and the 4-step AMD severity scale (65% vs. 63–75% or 67%), all without the need for retraining.

- Contributions: The study introduced a deep neural network with self-supervised NPID to predict AMD severity and classify referable AMD and advanced AMD without the need for labeled data. By utilizing the AREDS dataset and various classification methods, they demonstrated the flexibility of their approach in different AMD grading schemes. The NPID algorithm achieved balanced accuracies comparable to human ophthalmologists and supervised-trained networks in classifying AMD severity and referable AMD, showcasing its potential for unbiased and data-driven classification of ocular phenotypes related to AMD and other conditions.
- Limitations: The study’s reliance on a specific dataset may limit the generalizability of the results to other populations or clinical settings. Additionally, while the NPID algorithm demonstrated adaptability across different labeling schemes, further validation on diverse datasets is necessary to assess its robustness and applicability in real-world healthcare settings. Furthermore, the study’s emphasis on algorithmic performance highlights the need for comprehensive evaluation of clinical outcomes and patient impacts to ensure the effectiveness and safety of self-supervised learning-based diagnostic systems in clinical practice.

Thomas et al. [99] devised an algorithm for AMD diagnosis in retinal OCT images, employing statistical techniques, randomization, and RPE layer identification. The algorithm focuses on statistically categorizing AMD by utilizing the RPE layer and baseline to calculate Drusen height, indicative of disease severity. The methodology involves despeckling, RPE estimation, baseline estimation, Drusen height detection, and categorization. The evaluation was conducted on a publicly available dataset from Duke comprising 2130 slices from thirty participants. The proposed approach achieved an overall accuracy of 96.66%, surpassing comparable statistical techniques. An adaptive denoising method enhances RPE estimation by removing stray pixels under the RPE.

- Contributions: The study developed an algorithm for diagnosing AMD in retinal OCT images, focusing on statistically categorizing AMD severity using the RPE layer and baseline to calculate Drusen height. Their methodology, which includes despeckling, RPE estimation, baseline estimation, Drusen height detection, and categorization, achieved an impressive overall accuracy of 96.66%. By utilizing statistical techniques and randomization, their approach surpassed comparable methods, showcasing its potential for accurate AMD diagnosis and disease severity assessment in OCT images.
- Limitations: The study was conducted on a single publicly available dataset, which may limit the generalizability of the results to other datasets or clinical settings. Additionally, further validation on larger and more diverse datasets is necessary to assess the algorithm’s robustness and applicability in real-world healthcare scenarios. Furthermore, the study’s focus on statistical techniques may overlook potential nuances in AMD diagnosis that could be addressed by incorporating additional imaging modalities or clinical features.

Christiana et al. [100] proposed an automated AMD identification method using fundus images using DL algorithms. Image features were extracted with an Elite U Net model optimized with mustard sunflower optimization. Training and testing utilized the AREDS dataset, achieving high accuracy. Comparison with existing approaches indicates effectiveness in AMD identification. The clinical context presents challenges due to image variability, but the proposed method excels in distinguishing vessel bifurcations and crossings from AMDs with 98% accuracy and minimal error.

- Contributions: The study introduced an automated method for identifying AMD in fundus images using DL algorithms. Their approach utilized an Elite U Net model optimized with mustard sunflower optimization to extract image features, achieving high accuracy on the AREDS dataset. By comparing with existing methods, they demonstrated the effectiveness of their approach in accurately identifying AMD, particularly excelling in distinguishing vessel bifurcations and crossings from AMDs with 98% accuracy and minimal error.
- Limitations: The study was primarily conducted on a single dataset, which may limit its generalizability to diverse populations or clinical settings. Additionally, the clinical context of AMD diagnosis presents challenges due to image variability, suggesting the need for further validation on larger and more diverse datasets to assess the algorithm’s robustness across different imaging conditions and patient demographics. Furthermore, the reliance on DL algorithms may require careful consideration of computational resources and potential biases in training data to ensure reliable performance in real-world clinical practice.

Vaiyapuri et al. [101] introduced a multi-retinal disease detection model using the IDL-MRDD technique on fundus images. The model aims to classify images such as Normal, Hypertensive Retinopathy, AMD, DR, Glaucoma, Pathological Myopia, and Others. The IDL-MRDD model undergoes preprocessing, segmentation using AFA-SF, feature extraction with SqueezeNet, and classification via SSAE. AFA-SF-based multi-level thresholding enables accurate detection of infected zones. SqueezeNet generates feature vectors, while SSAE serves as the classifier. Evaluation on a benchmark multi-retinal disease dataset demonstrates superior performance with a maximum accuracy of 0.963 compared to state-of-the-art techniques.

- Contributions: The study proposed a multi-retinal disease detection model, the IDL-MRDD technique, for classifying fundus images into categories such as Normal, Hypertensive Retinopathy, AMD, DR, Glaucoma, Pathological Myopia, and Others. Their approach integrated preprocessing, segmentation using AFA-SF, feature extraction with SqueezeNet, and classification via SSAE.
- Limitations: The study was conducted on a specific benchmark dataset, which may limit its generalizability to other datasets or clinical scenarios. Additionally, further validation of diverse datasets representing various populations and clinical settings is necessary to assess the robustness and applicability of the model in real-world healthcare environments. Moreover, the computational complexity of the proposed technique, particularly involving multiple stages such as preprocessing, segmentation, feature extraction, and classification, may pose challenges in deployment, requiring consideration of computational resources and scalability for practical implementation.

Lee et al. [102] proposed two DL models, CNN-LSTM and CNN-Transformer, to predict the 2-year and 5-year risk of progression to late AMD by utilizing sequential information in longitudinal CFPs. LSTM and Transformer were utilized to capture this sequential data. The models were evaluated using data from the Age-Related Eye Disease Study (AREDS), one of the largest longitudinal AMD cohorts with CFPs. Results showed that the proposed models outperformed baseline models, which only considered single-visit CFPs, in predicting the risk of late AMD (AUC of 0.879 vs. 0.868 for 2-year prediction, and 0.879 vs. 0.862 for 5-year prediction).

- Contributions: The study proposed two DL models, CNN-LSTM and CNN-Transformer, for predicting the 2-year and 5-year risk of progression to late AMD by utilizing sequential information in longitudinal CFPs. Utilizing LSTM and Transformer architectures to capture sequential data, the models were evaluated using data from the AREDS, one of the largest longitudinal AMD cohorts with CFPs.
- Limitations: The study was conducted on data from a specific cohort (AREDS), which may limit their generalizability to other populations or clinical settings. Additionally, the complexity of DL models may pose challenges in interpretability and computational resources, requiring careful consideration in practical implementation. Furthermore, further validation on diverse datasets and external validation cohorts is necessary to assess the robustness and generalizability of the models in real-world clinical practice.

Fang et al. [2] utilized multiple CNN models in the ADAM challenge, including EfficientNet, DenseNet, Inception-ResNet, ResNeXt, SENet, Xception, Inception-v3, ResNet50, DenseNet101, Autoencoder with ResNet50 as backbone, ResNet-101, and EfficientNet-B4, to detect AMD into four categories using the ODIR dataset. The objectives of the challenge included detecting AMD, detecting and segmenting the optic disc, finding the fovea, and detecting and segmenting lesions from fundus images. The achieved results showed high performance with an AUC of 0.9714 and AUCs for Early AMD (0.9159), Intermediate AMD (0.9964), Advanced AMD dry (0.9914), and Advanced AMD wet (0.9917).

- Contributions: The study utilized multiple CNN models in the ADAM challenge to detect AMD and perform related tasks such as optic disc segmentation and fovea localization using the ODIR dataset. Their ensemble approach, combined with the incorporation of clinical domain information, led to high-performance outcomes. The study highlighted the importance of ensembling methods and the inclusion of clinical domain knowledge in enhancing DL model performance for automated solutions in ophthalmology.
- Limitations: The study was conducted on a specific dataset (ODIR), which may limit the generalizability of the findings to other datasets or clinical scenarios. Additionally, the reliance on ensembling methods and clinical domain information may introduce complexities in model interpretation and practical implementation. Furthermore, the study emphasized the importance of including clinical domain information for tasks such as AMD classification, but the impact of this information may vary depending on the specific task and dataset, requiring further investigation and validation on diverse datasets.

El-Den et al. [103] proposed an integrated model using color fundus images to scale input images and classify retinas as normal or belonging to various grades of AMD. The method consists of two phases: in the first phase, a custom auto-encoder-based model resizes input images and performs preprocessing as needed. The output is then fed into the second phase, where a pre-trained ResNet50 model classifies the images into normal retinas, intermediate AMD, GA, and wet AMD grades. The model aims to facilitate early identification of AMD grades for prompt treatment, potentially slowing down disease progression and reducing severity. The dataset used for this study was gathered from the University of Pennsylvania-sponsored Comparisons of AMD Treatments Trials (CATT). The model comprises a CNN classification network and an auto-encoder-based network for scale adaptation. Through experiments, the model achieved an accuracy, sensitivity, and specificity of 96.2%, 96.2%, and 99%, respectively, outperforming other models.

- Contributions: The study proposed an integrated model utilizing color fundus images to scale input images and classify retinas as normal or belonging to various grades of AMD. Through experiments, the model achieved impressive performance metrics outperforming other models.
- Limitations: The study was conducted on a specific dataset (CATT), which may limit the generalizability of the findings to other datasets or clinical contexts. Additionally, the reliance on color fundus images may overlook potential complementary information from other imaging modalities, such as OCT or angiography, which could further improve the accuracy of AMD classification. Furthermore, while the model achieved high accuracy, further validation on external datasets and diverse populations is necessary to assess its robustness and generalizability in real-world clinical settings.

Kadry et al. [104] developed an automated method for distinguishing between AMD patients and non-AMD patients using a DL scheme (VGG16 CNN) by merging deep features (DF) and handcrafted features (HF). The database included OCT images for assessment and fundus retinal images from the iChallenge-AMD database. The study encompassed data processing, manual feature extraction, DF extraction with VGG16, optimum feature selection using the Mayfly method, feature concatenation, binary classification, and validation. Handcrafted features such as local binary pattern (LBP), pyramid histogram of oriented gradients (PHOG), and discrete wavelet transform (DWT) were extracted from test images to enhance performance, and integrated with DF. The system was evaluated independently using CFP and OCT images. A SoftMax classifier verified detection performance, followed by a comparison of VGG16’s performance with VGG19, AlexNet, and ResNet50. In comparison to OCT images, which had accuracies ranging from 97.08% to 97.92%, the proposed VGG16 achieved an AMD detection accuracy of 97.08%, precision of 97.48%, sensitivity of 96.67%, specificity of 97.50%, and NPV of 96.69%.

- Contributions: The study developed an automated method for distinguishing between AMD and non-AMD patients using a DL scheme. The system demonstrated improved performance in AMD detection, especially with OCT images.
- Limitations: The study was conducted on a specific dataset, which may limit its generalizability to other datasets or clinical settings. Additionally, the comparison of VGG16’s performance with other DL models (VGG19, AlexNet, ResNet50) could be further expanded to include a broader range of architectures for a comprehensive assessment. Furthermore, the study’s emphasis on accuracy metrics may overlook potential biases or limitations in the dataset or algorithm, highlighting the need for further validation and evaluation in real-world clinical settings.

Kihara et al. [105] introduced a binary classification ViT method for identifying eyes with neovascular AMD (neMNV). Using a 6 × 6 mm scan pattern from swept-source OCT angiography (SS-OCTA), they processed 500 B-scans to generate an integrated face prediction map. This map was used with a segmentation model to compute prediction masks, distinguishing between Drusen and the double-layer sign (DLS) associated with neMNV. Annotated individual B-scans were employed, and graded by human evaluators to test the model. The algorithm achieved sensitivity, specificity, positive predictive value (PPV), and negative predictive value (NPV) of 82%, 90%, 79%, and 91%, respectively, with an area under the curve of 0.91. In comparison with human graders on the same set of 100 eyes, two junior graders independently identified DLSs in 24 out of 33 eyes with neMNV and correctly identified 56 out of 67 eyes without MNV, achieving 73%, 84%, 69%, and 86% for sensitivity, specificity, PPV, and NPV, respectively. The senior grader achieved 88%, 87%, 76%, and 94% for sensitivity, specificity, PPV, and NPV, respectively, detecting DLSs in 29 of 33 eyes with neMNV and correctly identifying absence in 58 of 67 eyes without MNV. The model demonstrated strong performance in detecting DLSs in eyes with late AMD, exhibiting substantial agreement with the senior human grader (κ=0.83, *p* < 0.001). Junior and senior human graders also showed high agreement (κ=0.68, *p* < 0.001) in their assessments.

- Contributions: For identifying neMNV, a deep learning model was created to differentiate the DLS linked to the neMNV from Drusen. The assessment showed that segmentation performance may significantly increase sensitivity and specificity in the final classification job and is consistent and dependable.
- Limitations: The model should only be used with structural SS-OCT scans with AMD because it was only evaluated on these scans. The model’s ability to identify tiny lesions is likewise rather limited. The size of the lesion and its performance are highly correlated. Small lesions are typically hard for the model to remove and hard to tell apart from Drusen from DLSs. If they include more cases in the training dataset, the performance could get better. Adding lesion size reweighting to their loss function while the ViT segmentation model is being trained is an additional strategy.

Xu, et al. [106] proposed a hierarchical vision transformer model for distinguish Normal AMD Dry AMD Wet AMD type-I MNV and type-II MNV using different CFP databases including WMUEH, ODIR, ADAM, and Ichallenge databases. Mixup and Cutmix are the two augmentation techniques they use. They employed “Center crop” and “Random Resized Crop” to extract the most pertinent portion of an image while resizing it to a particular size to increase the variety and unpredictability of the training dataset. In addition, they used “Random Horizontal Flip” to flip the image horizontally and produce a mirror image. Additionally, they included “Random Augment”, which automatically improves images by combining arbitrary rotation, translation, scaling, and shearing combinations. They used a technique called “Random Erasing”, which randomly erases reticular sections of the image to further enlarge the dataset. The classification model was motivated to concentrate on other areas and pick up more robust characteristics by excluding specific portions of the image. The hierarchical vision transformer’s general design was divided into four phases, each of which had a distinct function. A Swin Transformer block and a Linear Embedding module make up the first step. The remaining three steps, which comprised a Swin Transformer block and a patch merging module, have the same structure.

- Contributions: A deep learning model based on CFPs was suggested for the detection of differential AMD. Compared to ophthalmologists, DeepDrAMD is more efficient and lowers expenses. They compare traditional deep learning models such as CNN and MLP. The outcomes showed how well DeepDrAMD (vision transformer) worked in AMD identification, with a high AUC of 96.47% in the external Ichallenge-AMD cohort and 98.76% in the WMUEH test set.
- Limitations: While augmentation of data can greatly improve model performance, there are drawbacks as well. For example, over-augmentation can lead to overfitting and increase processing resource consumption. Appropriate data augmentation techniques must be chosen and used sparingly to balance data augmentation with computational resources and prevent overuse. DeepDrAMD may perform differently on various datasets and demographics, necessitating more testing and improvement. Furthermore, the model’s applicability to actual clinical situations and its incorporation into current healthcare systems need to be carefully considered and validated.

Dominguez et al. [107] extensively studied two families of deep learning models, CNNs and transformer architectures, for automatic diagnosis and severity grading of referable and non-referable AMD. They evaluated various scaling techniques and ensemble strategies to enhance the performance of convolutional architectures using fundus images from the UpRetina database. Among the eight CNNs (EfficientNet B3, EfficientNet v2, HRnet w32, Inception-v4, Inception-ResNetv2, ResNet50, ResnetRS50, and ResNeSt50) and nine transformer-based architectures (BeiT, CaiT, CoaT, DeiT, PiT, Swin, TNT, ViT, and Visformer), convolutional models consistently achieved an average AUROC of over 95%, nearly 80% mean κ value, and over 90% for other evaluation metrics. In contrast, transformer-based models performed less effectively, with mean κ values below 31% and mean AUROC values below 75% for AMD diagnosis. Their ensemble model for grading AMD severity achieved a mean accuracy (SD) of 82.55% (2.92) and a mean weighted κ coefficient (SD) of 84.76% (2.45). For diagnosing referable AMD, the models attained a mean F1-score (SD) of 92.60% (0.47), mean AUROC (SD) of 97.53% (0.40), and mean weighted κ coefficient (SD) of 85.28% (0.91).

Contributions: They have thoroughly examined many deep learning architecture families, training plans, and ensemble techniques to automatically identify AMD that is referable or non-referable and to provide a severity rating based on retinal fundus images. Convolutional-based designs outperform transformer-based architectures in this situation, despite the former’s encouraging findings when working with real images. Using a progressive scaling technique, where the models are trained with large images at first and then with smaller images, enhanced the performance of convolutional architectures. Binary models that are taught to grade AMD severity scales perform comparably to models that are just trained to diagnose referable versus non-referable AMD. Lastly, they may use test-time augmentation to further improve the performance of binary and multi-class convolutional-based models; however, other techniques such as the use of classical model ensembles or the cascade of models only serve to enhance the output of multi-class models.

Gholami et al. [108] utilized OCT image data from three research datasets to propose a federated learning (FL) approach for training deep learning models such as ResNet18 and ViT to identify AMD. They addressed domain shift concerns across institutions by integrating four domain adaptation algorithms. Their study demonstrated that FL techniques enabled competitive performance similar to centralized models, even with local models trained on subset data. The Adaptive Personalisation FL method consistently performed well across tests. The research underscored the effectiveness of simpler architectures in image classification tasks, particularly for privacy-preserving decentralized learning. Further investigation into deeper models and FL techniques was recommended for a comprehensive performance assessment. On test sets, ResNet18 achieved 94.58% ± 0.62 accuracy on the Kermany dataset, while ViT achieved 98.18% ± 0.55 and 99.11% ± 0.39 on the Srinivasan and Li datasets, respectively. These findings highlight FL’s critical role in healthcare analytics, ensuring patient privacy and enabling insights from distributed data.

- Contributions: Utilizing three different datasets, this study conducted a thorough set of tests to compare the efficacy of deploying DL models utilizing local, centralized, and FL approaches. Using ResNet18 and ViT encoders, the main goal was to classify OCT images into two binary categories: Normal and AMD. In addition, they incorporated four distinct DA techniques into the FL approach to address the widespread problem of domain shift.
- Limitations: They used an aggregation policy based only on a weighted average and a quite simple DL architecture throughout the training phase. Subsequent initiatives will investigate more complex aggregation policies. Despite these limitations, their findings enhance our understanding of FL techniques and offer priceless insights into the relative effectiveness of simpler structures for image classification tasks. We hope that exploring further FL techniques in future studies will provide more light on the subtleties of these models’ functionality. Another area that warrants attention is the distinct classification head, where amplitude normalization and intelligent weight aggregation strategy may increase FL network efficiency. Finally, exploring more complex multi-layer perceptron designs and extra transformer blocks in deeper models like ResNet50, ResNet101, or ViTs may change performance dynamics and provide new insights.

Retinal ViT is a revolutionary vision transformer design that integrates the self-attention mechanism into the area of medical image analysis employing fundus images, as reported by Wang et al. [109]. The last component of the proposed model was a feed-forward network design multi-label classifier. This classifier used a Sigmoid activation function with two layers. On the publicly available dataset ODIR-2019, the experimental results demonstrate that the suggested model performs better than state-of-the-art methods like ResNet, VGG, DenseNet, and MobileNet. The proposed method also outperforms the state-of-the-art algorithms in terms of F1 score (0.932 ± 0.06) and AUC (0.950 ± 0.03).

- Contributions: A unique vision transformer model was proposed to address the problem of multi-label categorization of retinal images. The suggested method may classify retinal images into a total of eight groups. This study demonstrated that transformer-based models can outperform CNN-based models in terms of performance. It should be noted that the suggested deep learning model’s utilized attention mechanism, which aims to identify the global correlations between distant pixels, may be responsible for this.
- Limitations: The unbalanced problem with the utilized dataset was not taken into account in this investigation. The DR (D), normal (N), and other abnormalities (O) categories in the ODIR-2019 dataset contain a much higher number of images than the other five classifications combined. Consequently, the performance of the suggested method may be impacted by the dataset’s unequal distribution. Second, the original vision transformer served as the basis for the given deep model. The key change made to the original vision transformer is located at the output layer to accommodate the need for multi-label classification. To obtain a more precise outcome, the inner workings of the vision transformer requirements should also be optimized. Lastly, the tests only used one particular dataset, which may not have been sufficient to demonstrate the generalizability of the suggested vision transformer design.

To automatically distinguish between AMD, DME, and normal eyes, Jiang et al. [13] suggested a CAD technique that uses a vision transformer to analyze OCT images from Duke University. The size of the images was changed in this investigation, and the results were then normalized. Following the model pruning, the classification accuracy remained unchanged and the recognition time reached 0.010 s. Vision Transformer after pruning showed superior recognition ability when compared to CNN image classification models (VGG16, Resnet50, Densenet121, and EfficientNet). The findings indicated that the vision transformer was a better substitute for making more accurate diagnoses of retinal disorders. Regarding the identification speed of each image, VGG16 and Vision Transformer outperformed other CNN models in this regard. After pruning, the vision transformer’s single image recognition speed was the quickest, taking only 0.010 s to complete. Its identification accuracy stays at 99.69% and it operates at a pace greater than any other model. When it comes to identification speed and accuracy, the vision transformer after trimming outperforms CNN models in identifying fundus OCT images.

Contributions: The study showed that vision transformers were the best at recognition and that their capacity to recognize objects quickly and accurately was unaffected by pruning, which indicates that there will be no missed diagnosis in practical applications. This research proposes a model that can more accurately represent the CAD of retinal disorders.

Table 6 summarizes the discussed DL-related studies.

#### 3.4.2. Machine Learning Techniques (Handcrafted Features)

Various ML algorithms are utilized to classify fundus images of AMD by identifying patterns and features within the data. These algorithms fall under the umbrella of ML, which encompasses techniques employed across diverse sectors to address various issues [10,11,110,111,112]. Learning algorithms can be categorized into two main domains based on the type of knowledge they acquire. Supervised learning involves explicit information or direct human involvement, while unsupervised or semi-supervised learning entails the system determining target-related patterns autonomously to varying degrees [33,113]. Over the past decade, computer vision applications like image classification and object identification have relied on handcrafted features. The complexity and quantity of these features have increased over time to better address the tasks at hand. To assess the performance of feature-based systems employing an ensemble of multiple features, various feature-based techniques are considered [114].

Fraccaro et al. [114] developed models to diagnose AMD using interpretable white box methods such as decision trees and logistic regression, as well as less interpretable black box methods like AdaBoost, random forests, and support vector machines (SVM). They utilized an Electronic Health Record (EHR) system in Genoa, Italy, collecting data during routine visits from patients with AMD, other retinal disorders, and healthy participants to detect AMD or other macular diseases. Patient demographics and clinical symptoms associated with AMD were recorded, including subretinal fibrosis, subretinal fluid, macula thickness, depigmentation area, subretinal hemorrhage, soft Drusen, retinal pigment epithelium defects/pigment mottling, and macular scar. The study included 487 patients (912 eyes), with SVM and decision trees performing at a mean of 0.90, and AdaBoost, logistic regression, and random forests at a mean performance of 0.92. Age and soft Drusen were identified as the most discriminating elements in diagnosing AMD by clinicians. They found a potential trade-off between performance and complexity, noting that the relationship between increased complexity and better performance is not always consistent. Even though a single variable could not lead to successful diagnosis, decision trees (average AUC of 90%) and logistic regression (average AUC of 92%) performed comparably to random forests.

- Contributions: The study provided the identification of age and soft Drusen as the most discriminating elements in diagnosing AMD and provided valuable insights for clinicians. Furthermore, they highlighted the potential trade-off between model performance and complexity, emphasizing that increased complexity does not always guarantee better performance.
- Limitations: The study revealed limitations in precisely identifying all consistent sets of diagnostic pathways, leading to occasional ambiguity in diagnostic decisions. Nonetheless, they that an automated system incorporating longitudinal data and new variables could help overcome these limitations, enabling the detection of different decision paths and facilitating timely diagnosis, particularly in distinguishing ambiguous patient subsets.

Fundus images are utilized to differentiate between normal and AMD classes, including Early AMD, Intermediate AMD, and Late AMD, as described by Mookiah et al. [12]. Features such as Pattern Occurrence (PO) and Linear Configuration Coefficients (CC) were extracted from these images. These features were then inputted into various supervised classifiers such as SVM. The features are sorted using the *t*-test *p*-value to categorize the normal and AMD classes. The system’s performance was evaluated using ten-fold cross-validation on Automated Retinal Image Analysis (ARIA) and Structured Analysis of the Retina (STARE) datasets obtained from Kasturba Medical Hospital in Manipal, India. The private dataset achieved an accuracy of 93.52%, sensitivity of 91.11%, and specificity of 95.93%, while the ARIA dataset achieved an accuracy of 91.36%, sensitivity of 92.18%, and specificity of 90.00%. The proposed approach yielded the best results for the STARE dataset using 22 significant features, with an average accuracy of 97.78%, specificity of 97.50%, and sensitivity of 98.00%.

- Contributions: By extracting features and employing *t*-test *p*-value sorting, the study achieved impressive classification accuracy. Evaluation on both ARIA and STARE datasets demonstrated high performance, with the STARE dataset yielding particularly excellent results, indicating the method’s potential for aiding clinicians in AMD diagnosis during mass eye screening programs.
- Limitations: The study has some limitations. Firstly, while the classification accuracy is high, the reliance on supervised classifiers like SVM may limit the model’s ability to generalize to unseen data or populations with different characteristics. Moreover, the study does not provide comprehensive insight into the interpretability of the extracted features, which could hinder clinicians’ understanding and trust in the diagnostic process. Finally, the proposed approach may require further validation and refinement before widespread adoption in clinical settings, considering factors such as scalability, cost-effectiveness, and integration with existing diagnostic workflows.

Phan et al. [111] proposed an automatic classification method for AMD to differentiate between Non-AMD, Early, Moderate, and Advanced stages in a telemedicine setting, ensuring robustness and reproducibility. Initially, a study was conducted using color, texture, and visual context in fundus images to identify the most critical aspects for AMD classification. The AREDS protocol was followed, employing a random forest and an SVM to evaluate feature importance and categorize images based on different AMD stages. The experiments utilized a database of 279 fundus photographs from a telemedicine platform. The results showed that local binary patterns in multiresolution were the most significant for AMD classification, irrespective of the classifier used. The technique exhibited promising performances across various classification tasks, with areas under the ROC curve for screening ranging from 0.739 to 0.874 and for grading from 0.469 to 0.685.

- Contributions: By utilizing color, texture, and visual context in fundus images and following the AREDS protocol, they identified local binary patterns in multiresolution as the most critical aspect for AMD classification, regardless of the classifier used. The technique demonstrated promising performances across classification tasks, showing reliability in image quality and superior discriminating power of LBP features.
- Limitations: The study’s reliance on a database of 279 fundus photographs from a telemedicine platform raises concerns about generalizability, emphasizing the need for further validation on a larger and more diverse sample size to fully validate their findings. Nonetheless, their proposed method represents a significant step forward in providing a reliable diagnostic tool for AMD in clinical settings and patient tracking.

Alfahaid et al. [40] classified OCTA images from different retinal layers as having wet AMD or normal control using a KNN classifier using rotation-invariant uniform local binary pattern texture characteristics calculated on 184 2D OCTA images (92 AMD, 92 healthy). Manchester Royal Eye Hospital donated images utilized in this investigation. Both the individual retinal layers and the collective layers underwent the categorization process. The algorithm performed admirably, as the mean accuracy of 89% for all layers combined and 98% for the outer, 89% for superficial, 94% for deep, and 100% for choriocapillaris layers.

- Contributions: The study’s most significant finding is that employing the local texture features based on the LBPsriu2 descriptor in the classification using OCTA images achieved outstandingly accurate results. The algorithm achieved promising results in processing all layers together and each layer separately. The highest accuracies were achieved with the outer and choriocapillaris layers since the deformities of blood vessels on these layers are very clearly observable. Four different retinal layers are tested in this study and the main purpose of using the various layers is to identify the layer that has the most discriminative information which describes the abnormal blood vessel patterns in wet AMD cases.
- Limitations: The dataset that was employed was not very large. Extensive experiments should be conducted to determine the ideal value of K neighbors, and its reliability for use in clinical ophthalmology should be assessed. It should also be tested on a sizable dataset to determine its efficacy and strength when a larger dataset is used, and its ability to determine the severity of the disease should be tested using a dataset that includes AMD cases at different stages.

Wang et al. [10] introduced a computer-aided diagnosis (CAD) model to differentiate between AMD, DME, and healthy macula using OCT data. The study utilized a publicly available OCT dataset from Duke University, Harvard University, and the University of Michigan. OCT images were analyzed using features based on linear configuration pattern (LCP) with correlation-based feature subset (CFS) selection. Various classification algorithms were employed, including neural network multi-layer perceptron (BP), quadratic programming-based sequential minimal optimization (SMO), SVM with polynomial kernel, logistic regression (LR), Naive Bayes, and Random Forest (RF) with J48 decision tree. The optimal model, based on the SMO approach, achieved an accuracy of 99.3% for each of the three sample classes. SMO surpassed BP and LR in specificity and sensitivity, ranking second in terms of AUC performance with a slight reduction. It excelled in AMD samples with an accuracy of 97.8% and ranked second in DME samples. LR performed well in detecting DME samples with an overall accuracy of 94.3%.

- Contributions: Utilizing publicly available datasets from reputable institutions, the study employed a comprehensive approach, utilizing features based on LCP with CFS selection. The study achieved remarkable accuracy with the SMO approach leading with impressive metrics for each of the three sample classes.
- Limitations: The high accuracy achieved may raise questions about overfitting, especially given the relatively small dataset utilized. Furthermore, while the SMO approach excelled in specificity and sensitivity, there was a slight reduction in AUC performance compared to other models. Additionally, while the study demonstrates the importance of selecting relevant features and enhancing feature extraction efficiency, the generalizability of the model to diverse datasets and clinical settings remains to be validated.

Nugroho et al. [115] compared the effectiveness of deep neural network features with handcrafted characteristics. The study utilized OCT images from the investigation by Kermany et al., categorizing scans into Normal, DME, Drusen (early stages of AMD), and CNV. Feature extractors included DenseNet-169, ResNet50, Local Binary Pattern (LBP), and Histogram of Oriented Gradient (HOG). A perceptron neural network without a hidden layer (logistic regression) served as the evaluated classifier, adequate for benchmarking with a linear classifier. Classifiers trained on features from deep neural networks performed the best, achieving 89% accuracy for ResNet and 88% for DenseNet, compared to 50% for HOG and 42% for LBP. The classifiers’ F1-score and LBP Feature Precision Recall were 0.23 and 0.42, respectively, while HOG features yielded 0.56 and 0.50, respectively. ResNet50 features scored 0.91, 0.89, and 0.89, and DenseNet-169 features scored 0.90, 0.88, and 0.88. Deep neural network-based techniques also demonstrated better results for underrepresented classes.

- Contributions: By comparing deep neural network features with handcrafted characteristics, the study provided valuable insights into the performance of various classifiers. Their outcome suggested that both deep neural network-based approaches provide superior feature sets for OCT image classification compared to HOG and LBP methods.
- Limitations: The study revealed limitations. While deep neural network-based methods showed superior performance, they may require larger datasets and computational resources for training compared to handcrafted feature extraction techniques like LBP and HOG. Additionally, despite the promising accuracy rates, further validation on diverse datasets and clinical settings is necessary to assess the generalizability of the findings. Moreover, the study’s focus on OCT images may limit its applicability to other imaging modalities or multi-modal approaches, which could provide complementary information for disease diagnosis and monitoring.

Hussain et al. [11] proposed a classification approach for automatically identifying individuals with DME or AMD using retinal characteristics from Spectral Domain Optical Coherence Tomography (SD-OCT) images. SD-OCT images were obtained from four sources: Tian et al., Duke University, New York University (NYU), and Centre for Eye Research Australia (CERA). Retinal parameters such as retinal thickness and distinct retinal layers, as well as the volume of diseases like Drusen and hyper-reflective intra-retinal spots, were utilized in the classification process. Ten clinically significant retinal characteristics were automatically extracted for classification by segmenting individual SD-OCT images. The efficacy of the retrieved characteristics was assessed on 251 participants (59 normal, 177 AMD, and 15 DME) using various classification techniques, including Random Forest. Fifteen-fold cross-validation tests were performed for three phenotypes—DME, AMD, and normal cases—using these datasets. The Random Forest classification method achieved an accuracy of over 95% for each dataset, and when trained as a two-class problem consisting of normal and pathological eyes, the system yielded an accuracy of over 96%. Each dataset also yielded an area under the receiver operating characteristic curve (AUC) value of 0.99.

- Contributions: Their approach utilized retinal parameters obtained from multiple sources and utilized advanced segmentation techniques to extract ten clinically significant retinal characteristics for classification. Their findings revealed a strong correlation between the thicknesses of the layers and the projected diseases.
- Limitations: The approach may have limitations, particularly in cases of severe diseases where even state-of-the-art segmentation techniques may miss retinal layers. This suggests the need for further investigation and refinement, particularly in enhancing sensitivity to improve outcomes in such scenarios.

Li et al. [23] introduced three integration frameworks, including the innovative ribcage network (RC Net), which effectively combines manually crafted features with deep learning approaches such as VGG, DenseNet, and Xception. By demonstrating the effectiveness of adding handcrafted features like Gabor and scale-invariant feature transform (SIFT) in improving deep network classification accuracy, the study highlights the potential for enhancing DNN performance, particularly in scenarios with limited training data. The experimental results indicated that RC Net outperforms other integration techniques, achieving impressive accuracy, sensitivity, and specificity metrics. Notably, the study found that early integration techniques perform comparably to late integration methods but with reduced computational complexity, suggesting practical advantages in terms of computing time and model parameters. Additionally, the use of dense blocks and a sum operation in RC Net instead of convolutional blocks and concatenation contributes to parameter efficiency and network performance enhancement.

- Contributions: The work significantly advanced eye disease categorization by introducing integration frameworks that combine deep learning and handcrafted features, notably proposing the RC Net for feature integration, which outperformed other techniques utilizing dense blocks and a sum procedure, RC Net achieved high accuracy, sensitivity, and specificity. The study emphasized the importance of incorporating handcrafted features to enhance DNN performance, especially with limited training data. Additionally, it highlighted the efficiency of early integration techniques in reducing computing time and parameters.
- Limitations: The retrospective nature of the OCT image dataset may introduce biases, and the generalizability of the findings to other populations or imaging modalities needs further exploration. Moreover, while the study demonstrates the efficacy of integrating handcrafted features, the specific selection and combination of these features may require further validation across diverse datasets and disease categories. Overall, Li et al.’s work provides valuable insights into the integration of deep and handcrafted features for improved disease categorization, paving the way for further advancements in medical image analysis.

Govindaiah et al. [110] proposed a method to improve prediction probabilities by combining five different ML and statistical algorithms (Random Forest, Naïve Bayes, Logistic model tree, Simple Logistic, Multilayer Perceptron) using data from the AREDS study, which included 566 participants, in each of the six models. Two independent subsystems were included in each prediction model to predict the late wet AMD and late dry AMD categories. ML models were developed to predict late AMD and category in a single visit, in 2, 5, and 10 years, using different combinations of genetic, sociodemographic (S-D)/clinical, and retinal imaging data. In their investigation, they employed a wide range of factors, many of which might be challenging to find in a related study conducted elsewhere for external validation. They used the “NAT-2” dataset from an AMD study to test a model based on retinal images, with encouraging outcomes. The inclusion of genetic, clinical, and sociodemographic variables during model training may enhance the overall efficacy of late AMD prediction models. Each model’s performance was evaluated using 10-fold cross-validation due to the limited dataset. The model yielded 72.9% accuracy, with 73.8% sensitivity and 72.7% specificity.

- Contributions: The study demonstrated the potential for developing dependable prediction systems for late AMD in real-world settings. Furthermore, the finding that imaging alone can accurately predict late AMD for shorter timeframes without genome sequencing is noteworthy, indicating practical implications for early detection and intervention.
- Limitations: The dataset used for training and evaluation is relatively small, which may affect the generalizability of the findings to broader populations. Additionally, while the models demonstrated promising accuracy, sensitivity, and specificity, there is room for improvement, particularly in predicting late AMD for longer durations and in distinguishing between dry and wet forms. Moreover, the reliance on retrospective data from the AREDS study and the NAT-2 dataset may introduce biases and limit the applicability of the models to more contemporary datasets. Finally, the study acknowledges the potential for enhancing predictions with spectral-domain optical coherence tomography (SD-OCT), suggesting avenues for future research to incorporate advanced imaging techniques for improved accuracy and reliability.

Table 7 summarizes the discussed ML-related studies.

#### 3.4.3. Retinal Experts Classification

One of the main causes of permanent vision loss is AMD, which can be treated with injections of anti-vascular endothelial growth factor (VEGF) medicines to treat macular neovascularization (MNV). Several studies demonstrated the superiority of OCTA over other imaging methods to identify MNV (macular neovascularization) in eyes with macular degeneration.

For instance, Corvi et al. [43] assessed the efficacy of OCTA in comparison to OCT, indocyanine green angiography (ICGA), and fluorescein angiography (FA), in detecting MNV in eyes suffering from atrophy. Multimodal imaging using FA, ICGA, structural OCT, and OCTA was performed on eyes with MNV and atrophy (also known as macular atrophy or MA) attributable to AMD, as well as AMD eyes with GA without MNV. Senior retina experts used all imaging modalities to determine the existence of MNV, which was regarded as the gold standard reference. Two professional readers then independently assessed each unique imaging modality for the existence of MNV. On the specially designed OCTA slab, the morphologic properties of the MNV were assessed. They enrolled 21 patients with GA alone and 21 with MA+MNV. High specificity (95.2%) and sensitivity (95.2%) were demonstrated by the manual segmentation on OCTA, which enabled the identification of the MNV in 4.7% of eyes with GA and in 95.2% of eyes with MA+MNV. In 57.1%, 52.3%, and 66.7% of eyes with MA+MNV and in 14.2%, 9.5%, and 42.8% of eyes with GA, FA, ICGA, and OCT identified MNV. For FA, the corresponding values were 85.7% and 57.1%, for ICGA, 90.5% and 52.4%, and for OCT, 66.7% and 57.1%. Their approach of using data from various modalities and having this assessed by a senior specialist is a fair choice in the lack of a histopathological reference.

- Contributions: The study demonstrated the ability of OCTA to detect MNV in eyes with atrophy compared with fluorescein angiography (FA), ICGA, and OCT. This study has several strengths including its prospective design, use of multiple graders, and a masked grading process. The use of deeper penetrating SS-OCTA is also a strength. They also evaluated the morphological features of MNV associated with MA.
- Limitations: There are some limitations to this study. To start, the sample size is modest. On the other hand, it might not happen very often for new MNV to grow in an eye that has previously experienced atrophy. This study’s cohort was put together with a 50/50 mix of patients with and without MNV, which is another important drawback. Because of this, the group is “selected” and does not represent the overall clinic population of atrophy-affected eyes; as such, the results of the sensitivity, specificity, PPV, and NPV should be interpreted accordingly. A further plausible constraint on the research is the “gold” or reference standard. Histology would have been the perfect gold standard, but that is not feasible.

A procedure to assess and harmonize a cutting-edge nomenclature for reporting neovascular AMD data was developed by Spaide et al. [116]. For neovascular AMD, they developed a consensus categorization. The elements of neovascular AMD were identified and divided into groups. The effects of macular neovascularization on disease were outlined. Three key documents were created: an explanation of AMD, a classification of the many forms of neovascular AMD, and a framework for a consensual nomenclature system. Creating a common set of terminology will make it easier to compare various patient groups and research projects. The framework that has been given is easily updated and modified, a process that is expected to happen periodically. The development of OCT and OCT angiography, in particular, which have allowed for extensive three-dimensional investigation of the vascular anatomic and topographic features inside neovascular AMD lesions, has proven essential in the suggested categorization. The standardization of AMD investigation and reporting will be enhanced by the use of recognized categories and nomenclature. Increased nomenclature clarity will improve AMD study reporting and comparability. Increased terminology accuracy will improve AMD study comparability and reporting.

- Contributions: In addition to helping to enhance standardization and communication between researchers, doctors, and patients, the classification offers a way to group neovascularization and other lesion components in neovascular AMD. This new categorization makes research in the future more thorough and broadly applicable. It also aids in bringing definitions from clinical investigators and reading centers into harmony. The classification’s broad structure for other data to be added, such as analyses of the definitions’ repeatability and accuracy, to further improve it.
- Limitations: This method has been criticized because cRORA and MNV are distinct illnesses with distinct pathophysiologies, and as such, they should not be compared. This staging system’s foundation came from a time when late AMD was associated with an exceptionally high risk of serious visual impairment. For example, if fluorescein angiography revealed a neovascular lesion, the patient was usually at high risk of significant vision loss. Predicting the risk of late disease progression rather than visual function was the aim. Patients with MNV are likely to have stabilization or improvement in their visual acuity with current treatment; thus, grading the disease’s severity—an evaluation of the entire impact of the illness on an organ, including both reversible and permanent effects—becomes necessary.

Optical coherence tomographic (OCT) angiography was used to assess eyes with AMD and high-risk features for CNV in Palejwala et al. [117]. They demonstrated the usefulness of OCTA for early detection of CNV and identified early CNV (type I) in their series, which was challenging to identify using traditional FA and SD-OCT. Two (6%) of the 32 eyes had Type 1 CNV according to OCT angiography. Neither fluid on OCT nor leakage on fluorescein angiography was linked to the lesions. When nonexudative CNV lesions are present, they are difficult to distinguish with fluorescein angiography and OCT. The Casey Eye Institute’s retina clinics at Oregon Health and Science University in Portland, Oregon, served as a source for study participants.

- Contributions: They assessed eyes with high-risk features for CNV and AMD. Nonexudative CNV lesions are challenging to distinguish using FA and OCT; however, OCTA can determine their existence.
- Limitations: The data set used in this study is small, It should be tested on a sizable dataset to determine its efficacy and strength when a larger dataset is used.

Coscas et al. [118] evaluated 80 eyes with wet AMD using OCTA scanning and found several CNV patterns. Their group further received routine multimodal imaging, which was based on FA, spectral domain OCT (SD-OCT), and ICGA, to see if treatment was required. They utilized 80 exudative AMD eyes that were identified with various forms of CNV: polyps linked to AMD, mixed Type I and II, Type I, Type II, and retinal angiomatous proliferation. The results of the OCTA were utilized to distinguish between two distinct patterns of CNV and the investigation was carried out at the Odeon Ophthalmology Centre in Paris, France. The results of several multimodal imaging and OCTA analyses were then compared to assess any potential relationships between the CNV aspect of OCTA and the treatment choice. Conventional multimodal imaging revealed a CNV lesion in 58 eyes (72.5%) classified as Group A (requiring treatment). Fifty-nine eyes (73.7%) had an OCTA lesion classified as Pattern I, while 21 eyes (26.3%) had a Pattern II lesion. The Pattern I CNV on OCTA and the cases Group A on conventional multimodal imaging corresponded with 94.9% accuracy. Additionally, a 90.5% correlation between Group B (non-treatment-requiring) cases on conventional multimodal imaging and Pattern II CNV on OCTA was calculated.

- Contributions: To help patients with exudative AMD make treatment decisions, they compared OCTA with OCT, FA, and ICG. While OCT displays fluid buildup and its changes and fluorescein angiography is still the gold standard for detecting leakage, OCTA provides noninvasive monitoring of the CNV and supports every treatment decision made during the follow-up. Based on the availability of both functional and morphological information from a single OCT scan, the study proposes that OCTA is a valuable tool for noninvasive monitoring of the state of CNV in AMD. This kind of observation might be useful in directing treatment choices and assessing how well CNV responds to medication.
- Limitations: The use of a prototype device, the small number of patients, the lack of a three-dimensional reconstruction of the CNV, and the qualitative assessment of OCTA and multimodal imaging results are the primary limitations of this work.

In their publication, de Carlo et al. [119] detailed the features, sensitivity, and specificity of CNV detection by OCTA. Participants: 61 participants total—48 eyes of 43 people with CNV and 24 eyes of 18 subjects without CNV. There were 72 eyes in total. New England Eye Centre patients scanned with the OCTA system were evaluated. Patients whose OCTA revealed CNV were assessed to determine the features of the CNV, including appearance, size (measured using the largest linear dimension), and the existence of intraretinal and subretinal fluid. To determine the sensitivity and specificity of OCTA in identifying CNV using FA as ground truth, a second cohort of patients who had same-day OCTA and fluorescein angiography (FA) for suspected CNV was also analyzed concurrently. Thirty-one of the 48 eyes in the group exhibited CNV linked to neovascular AMD. When comparing OCTA to FA, the specificity of CNV detection was high (91%) while the sensitivity was low (4/8).

- Contributions: By using OCTA, a doctor may noninvasively visualize CNV and potentially detect and guide treatment for the condition. When compared to FA, the specificity of CNV detection using OCTA appears to be high.
- Limitations: This study’s modest sample size and primarily cross-sectional design are its limitations. Furthermore, in certain cases, the automated prototype program did not allow for the segmentation to eliminate choriocapillaris since the user could not manually modify the curvature lines designating the region of interest that was automatically recognized.

Table 8 summarizes the discussed experts-related studies.

### 3.5. Model Performance Evaluation Metrics

Three fundamental criteria are employed: accuracy, specificity, and sensitivity [120]. These metrics may also be applied in the multi-classification situation by extending the binary class to several classes [121]. These metrics play crucial roles in evaluating the effectiveness and reliability of algorithms and models in medical image analysis [122]. Table 9 summarizes various performance metrics commonly used in fundus image analysis.

## 4. Discussion and Summary

This study demonstrated that the use of ML and DL algorithms in conjunction with the OCT or CFP imaging method, either alone or in combination, yields promising results in the diagnosis of various stages of AMD. OCT and OCTA are more precise for many entities viewed with these technologies and can reveal anomalies not detected by color fundus photography or fluorescein angiography. They are not replaced by using OCT and OCT angiography; rather, these additional imaging modalities enhance the former and offer more information to enhance categorization options.

This study included a detailed examination of the various ML and DL approaches that have been recently employed for AMD diagnosis, as well as a comparison of the efficacy of fundus and OCT imaging methods. The results emphasize the importance of CFP and OCT imaging in assessing the risk of AMD in its early, middle, and late phases. The more than 250 million AMD patients who are expected to be diagnosed globally by 2040 would benefit greatly from improved triaging, counseling, and care because of the use of OCT and CFP imaging in AMD diagnosis. As of right now, CFP is more common than OCT imaging. Nevertheless, we did see a little increase in the ability to predict the development of late AMD when utilizing both OCT imaging and CFPs, indicating that the automatically determined risk from OCT imaging may provide some additional benefit to CFP. As a result, combining the two approaches might still help enhance illness progression prediction. To enhance the prognosis of AMD development, there are still more chances to take advantage of the abundance of data on OCT imaging.

An important result of those investigations is that the phases of AMD are now well classified. The accuracy and consistency of classifying AMD into GA, intermediate AMD, normal, and wet AMD categories have improved via extensive testing and research. This improved accuracy is a vital first step in enabling patient-appropriate treatment plans. These investigations have also shed light on complex patterns and correlations seen within fundus images. These patterns make it possible to recognize the subtle signs of various stages of AMD, which can help with early detection and therapy. The CAD system facilitates improved patient care by automating the AMD diagnostic process and reducing interobserver differences.

Several existing problems in this field should be noted in this study. The CAD system’s generalizability may be impacted by variances in image quality and demographic representation, among other factors that impact data quantity and quality. AMD stage labeling on fundus images may cause inter-observer variability, which might affect the assessment and training of ML models. Furthermore, a few studies mainly concentrate on the general classification of AMD stages, leaving out more specific subtypes. Verifying practical usefulness in various therapeutic contexts requires external evaluation. To responsibly implement the CAD system, clinical integration, regulatory permissions, and ethical considerations must be taken into consideration. Transparency is lacking in DL models, and clinical expertise should always be complemented by the CAD system. The accuracy of the categorization of AMD at different stages, particularly late stages, is the topic’s largest issue. There is not a gold standard for evaluating AMD categorization quality. We want to create a classification model for AMD in the future and provide fixes for these issues. We have already introduced a new promising method for AMD classification called concentrated ML-based classification system for AMD diagnosis using fundus images [18].

The results of this survey may be summed up as follows:

- The gold criteria for diagnosing wet AMD are CFP and OCT.
- The two main techniques for identifying and tracking dry AMD are CFP and OCT can further offer supplementary data. OCT is utilized to detect and track anomalies associated with AMD, including pseudoDrusen, Drusen deposits, subretinal fluid, RPE detachment, and choroid NV.
- AI components have shown an exceptional ability to aid with automated early identification, diagnosis, and staging of AMD disorders using several medical image modalities.
- Conventional ML techniques differ in the imaging modalities they employ, the features they extract, and the classifiers they employ. The literature has employed fundus imaging and OCT for AMD diagnosis, staging, and detection. OCT and fundus imaging have been employed for AMD staging, diagnosis, and detection.
- DL techniques, such as CNNs, have been applied to achieve increased performance and set the standard for future years in the automated diagnosis, staging, and detection of AMD disorders. OCT and fundus imaging have been employed for AMD staging, diagnosis, and detection.

## 5. Future Research Directions

In the rapidly evolving landscape of retinal image analysis, future research endeavors can explore several avenues to further enhance the understanding and application of machine learning and deep learning methodologies. One potential area of focus is the expansion of the discussion on different modalities. Delving deeper into the integration of various modalities beyond retinal fundus images, such as OCT scans and angiography, can provide a comprehensive understanding of retinal pathology and improve diagnostic accuracy.

Additionally, future studies could explore complementary non-AI approaches to offer alternative perspectives and insights. Investigating traditional image processing techniques, statistical modeling, and expert systems alongside AI-driven methodologies can contribute to a holistic approach to retinal disorder diagnosis.

Furthermore, examining the practical implementation of AI systems in clinical settings is crucial for their adoption and efficacy. Future research should focus on evaluating the scalability, usability, and integration of AI models into existing healthcare infrastructure, addressing concerns related to regulatory compliance, data privacy, and clinician acceptance.

Diversifying the datasets used for training and evaluation is another crucial direction for future research. Researchers can explore publicly available datasets, collaborate with healthcare institutions to acquire diverse data sources and investigate data augmentation techniques to mitigate biases and improve model performance across different populations and demographics.

Lastly, while AMD is a significant focus of current research, exploring the applicability of ML and DL techniques to other retinal diseases, such as glaucoma, can broaden the scope of impact in ophthalmology. Investigating disease-specific features, biomarkers, and diagnostic algorithms can aid in early detection and personalized treatment strategies for a wider range of ocular conditions.

## 6. Conclusions

Automated techniques for diagnosing eye-related disorders are increasingly essential, particularly considering the shortage of doctors compared to the growing patient population. With the abundance of OCT and color fundus images containing a plethora of eye-related conditions, there is immense potential for medical image analysis studies. The utilization of various ML/DL models for automated disease diagnosis is a promising avenue. This review has provided a systematic approach to understanding the latest ML and DL techniques in ocular disease diagnosis, focusing on AMD. The significance of the training dataset cannot be overstated, as it significantly impacts the performance of DL models. Therefore, a comprehensive description of publicly available fundus image datasets has been provided, acknowledging the variability in image quality and environmental conditions across different datasets. While some datasets offer high-quality images captured under controlled conditions, others encompass images taken in diverse environmental settings. It is crucial to curate datasets that reflect real-world scenarios to develop reliable models applicable in clinical settings. Moreover, preprocessing techniques such as contrast enhancement, color space transformation, and image augmentation have proven beneficial in improving the performance of ML and DL models by facilitating the extraction of disease-relevant features during training. Recent studies have explored various backbone models, including Inception, VGG, ResNet, and Basic CNN, for AMD classification tasks. However, these models have typically served as foundational frameworks, with additional learning paradigms like active learning, multitask learning, transfer learning, and group learning being investigated to further enhance model performance and provide precise diagnoses.

## Figures and Tables

**Table 1:** Tabular depiction of AMD fundus image stages showcasing a comparison along with visual samples.

No AMD (Normal)	Early	Intermediate AMD	Advanced Dry AMD	Advanced Wet AMD
Refers to images showing no signs of AMD-related changes, often depicting individuals without clinical evidence of AMD or those in early stages of the disease with minimal abnormalities, such as a small number (5–15) of small (<63 μm) or absent Drusen without pigment alterations.	Early AMD clinically presents with Drusen and abnormalities in the retinal pigment epithelium (RPE). It may involve one or both eyes, showing pigmentary changes, a few small Drusen, and/or intermediate-sized (63–124 μm) Drusen.	Intermediate AMD lies between early and advanced stages, characterized by pigmentary changes and Drusen, small yellow deposits beneath the retina. It may include one large druse (>125 μm), one extensive intermediate-sized druse (20 soft or 65 hard without any soft), and/or GA excluding the macula in one or both eyes.	GA is marked by the gradual loss of RPE cells in specific macular areas, forming well-defined atrophic patches. It results in progressive central vision loss and is a major cause of significant visual impairment in AMD patients.	Wet AMD is an advanced and severe form of the disease, characterized by the growth of abnormal blood vessels beneath the retina. These vessels can leak fluid or blood, causing retinal damage and rapid vision loss if untreated. It commonly results in sudden and severe central vision impairment.
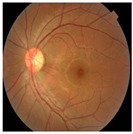	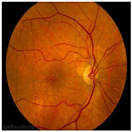	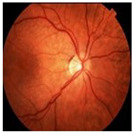	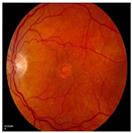	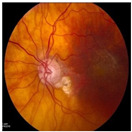

**Table 2:** Tabular comparison between the different imaging modalities.

Modality	When to Use	Cost	Pros.	Cons.
Color Fundus Photographs (CFP)	Screening for retinal abnormalities	Low cost	Detailed images of the back of the eye.	Inconsistent pigmentation, inability to resolve fundus depth.
Optical Coherence Tomography (OCT)	Evaluation of retinal disorders	High cost	High-resolution cross-sectional images of the retina.	Limited by medium opacities, operator skill required.
Optical Coherence Tomography Angiography (OCTA)	Detection of microvascular alterations	Moderate cost	Non-invasive, three-dimensional imaging of blood vessels.	Limited normative data, affected by media opacity.
Fluorescein Angiography (FA)	Identifying and categorizing CNV	Moderate cost	Evaluation of retinal and choroidal circulation pathology.	Invasive, requires dye injection, limited to 2D imaging.
Indocyanine Green Angiography (ICG)	Visualization of choroidal circulation	Moderate cost	Deeper penetration into the retina, clearer images of choroidal veins.	Invasive, limited diffusion, requires skilled interpretation.

**Table 4:** Summarization of the pre-processing techniques for fundus images utilized in diagnosing retinal disease [31,69,70,72].

Pre-Processing Technique	Description	Complexity	Effectiveness	Robustness	Ease of Implementation
Color Space Transformation	Enhances DL model performance by selectively utilizing a single color channel from the RGB Channels, often removing the green channel to eliminate visually rich information, particularly in high contrast images	Low	Moderate	High	High
Cumulative Distribution Function (CDF)	Simplifies understanding of image features and pixel intensity distribution through a cumulative probability distribution, aiding in identifying areas of interest	Low	High	Moderate	Moderate
Noise Removal	Eliminates unwanted noise using various denoising algorithms such as non-local means denoising, median filters, and Gaussian filters	Moderate	High	High	Moderate
Contrast Enhancement (CLAHE)	Widely used approach for enhancing contrast and addressing over-amplified contrast in certain pixel portions, improving the quality of fundus images for analysis	Moderate	High	High	High
Segmentation Mask	Utilizes binary masks to isolate regions of interest (ROIs) within fundus images, improving diagnostic accuracy by selecting specific regions for analysis while excluding background noise	Moderate	High	High	Moderate
Contour Analysis	Essential for fine-tuning ROIs and locating object boundaries in images, providing attributes like centroid, area, and perimeter for object modification and determination	Moderate	High	Moderate	Moderate
Augmentation	Balances image datasets through techniques like rotation, translation, flipping, and rescaling, enhancing model robustness and performance	Low	High	High	High
Cropping and Extracting ROI	Isolates significant areas within images for analysis, reducing unnecessary learning effort during model training	Low	Moderate	High	High
Histogram Equalization	Enhances overall contrast in fundus images, making background pixels stand out and improving image clarity.	Low	Moderate	Moderate	High
Resized Image	Maintains consistency across the dataset by resizing images to standard dimensions	Low	Low	High	High
Enhanced Image (Improving Contrast)	Lowers noise and enhances contrast in images, improving overall image quality	Moderate	High	Moderate	High

**Table 5:** Summary of the machine and deep learning segmentation approaches.

Study	Method	Dataset	Segmentation Type	Performance	Modality
Yan et al. [74]	Encoder-decoder Network	STARE, DRIVE	Drusen segmentation	Accuracy: 97.13%, Sensitivity: 92.02%, Specificity: 97.30%	CFP
Pham et al. [67]	DeeplabV3,U-Net	Kangbuk Samsung hospital, STARE	Drusen segmentation	Accuracy: 0.99, 0.981%, Sensitivity: 0.662, 0.588%, Specificity: 0.997, 0.991% Dice Score: 0.625, 0.542	CFP
Chen et al. [75]	Faster R-CNN	RETOUCH	Cysts/fluid	Accuracy: 0.665%, Dice Score: 0.997	OCT
Schlegl et al. [66]	FCN	1200 volumes OCT	Cysts/fluid	Sensitivity: IRC 0.84, SRF 0.81%, Precision: IRC 0.91, SRF 0.61, Area under the curve (AUC): IRC 0.94, SRF 0.92	OCT
Sappa et al. [76]	RetFluidNet	124 volumes OCT	Cysts/fluid	Accuracy: IRF 80.05, PED 92.74, SRF 95.53%, Dice Score: 0.885	OCT
Kang et al. [77]	U-Net	RETOUCH	Cysts/fluid	Accuracy: 0.968%, Dice Score: 0.9	OCT
Liu et al. [78]	FCN	RETOUCH	Cysts/fluid	Dice Score: 0.744	OCT
Tennakoon et al. [79]	U-Net	RETOUCH	Cysts/fluid	Dice Score: 0.737	OCT
Diao et al. [80]	CM-CNN, (CAM-UNet)	Heidelberg Engineering, Germany	Drusen and CNV lesions	Dice Score: 77.51%	OCT

**Table 6:** Tabular summary of the DL-related classification approached discussed in the current review in AMD diagnosis.

Study	Method	Dataset	Classification	Performance	Modality
Karri et al. [19]	Transfer learning with Inception network	DHU database	Dry AMD, DME, Normal	Avg. Accuracy: 99% (Normal), 86% (DME), 89% (AMD)	OCT
Rasti et al. [20]	Multi-scale Convolutional Mixture of Expert (MCME) ensemble model	NEH, DHU dataset	Dry AMD, DME, Normal	Accuracy: 98.33% (DHU), 99.39% (NEH)	OCT
Philippe et al. [92]	DL with transfer learning on fundus images	AREDS database	Disease-free/early stages, Referable intermediate/advanced stages	Accuracy: 88.4–91.6%, AUC: 0.94–0.96, human performance levels (accuracy = 90.2κ = 0.800)	CFP
Thakoor et al. [41]	Deep learning (CNNs)	faculty at Columbia University Medical Centre	NV-AMD vs.non-NV-AMD vs. healthy	Accuracy: 77.8% (NV-AMD vs. non-NV-AMD vs. healthy), 93.4% (NV-AMD vs. healthy),	OCTA
Tan et al. [1]	Custom-designed CNN	KMC dataset	Dry AMD, Wet AMD, Normal	Accuracies: 91.17% (blindfold), 95.45% (ten-fold CV)	CFP
Jain et al. [95]	LCDNet for binary classification	ML data repository, Retinal Institute dataset	Diseased or Healthy	Accuracy: 96.5–99.7%	CFP
Islam et al. [96]	CNN-based method	ODIR dataset	Eight ocular disorders	AUC: 80.5%, κ: 31%, F-score: 85%	CFP
Li et al. [21]	DL with VGG16	Private retinal OCT image database	CNV, Normal AMD	AUC: 100%, Accuracy: 98.6%, Sensitivity: 97.8%, Specificity: 99.4%	OCT
Bhuiyan et al. [97]	CNNs with AREDS dataset	AREDS dataset	Referable AMD classification	Predictions based on image probabilities	CFP
Zapata et al. [61]	CNN-based classification algorithm	Optretina dataset	AMD Disease vs. No disease	Accuracy: 86.3%, AUC: 0.936, Sensitivity: 90.2%, Specificity: 82.5%	OCT, CFP
Bulut et al. [93]	Xception model with transfer learning	Akdeniz University Hospital dataset	Retinal defects	Accuracy: 82.5–91.39%	OCT, CFP
Xu et al. [94]	DL approach with ResNet50	Peking Union Medical College Hospital dataset	PCV, Wet AMD, Dry AMD, AMD	Accuracy: 87.4%, Sensitivity: 88.8%, Specificity: 95.6%	CFP
Yellapragada et al. [98]	Deep neural network with self-supervised NPID	AREDS dataset	AMD severity, Referable AMD, Advanced AMD	Balanced accuracies comparable to ophthalmologists	OCT, CFP
Thomas et al. [99]	Algorithm for AMD diagnosis in retinal OCT images	Duke dataset	AMD categorization	Overall Accuracy: 96.66%	CFP
Christiana et al. [100]	Elite U Net model	AREDS dataset	AMD identification	Accuracy: 98%	OCT
Vaiyapuri et al. [101]	IDL-MRDD technique with fundus images	Benchmark multi-retinal disease dataset	Multi-retinal disease detection	Max accuracy: 96.3%	CFP
Lee et al. [102]	CNN-LSTM and CNN-Transformer	AREDS dataset	2-year and 5-year risk prediction of late AMD	AUC: 0.879 (2-year), 0.879 (5-year)	CFP
Fang et al. [2]	Multiple CNN models in ADAM challenge	ODIR dataset	AMD detection into four categories	AUC: 0.9714, High performance in classification tasks	CFP
El-Den et al. [103]	Integrated model with auto-encoder and ResNet50	CATT dataset	Normal, Intermediate AMD, GA, Wet AMD	Accuracy: 96.2%, Sensitivity: 96.2%, Specificity: 99%	OCT
Kadry et al. [104]	DL (VGG16 CNN) + Handcrafted features (LBP, PHOG, DWT)	OCT and fundus retinal images from iChallenge-AMD database	AMD detection	Accuracy: 97.08%	CFP
Kihara et al. [105]	ViT	Carl Zeiss Meditec, Inc.	Normal, AMD patients with and without neMNV	Sensitivity = 82%, specificity = 90%, PPV = 79%, and NPV = 91%, AUC = 0.91	OCTA
Xu et al. [106]	Hierarchical vision transformer	WMUEH, ODIR, ADAM	Normal AMD DryAMD WetAMD type-I MNV type-II MNV	AUC of 98.76%(WMUEH test set), 96.47% (iChallenge set)	CFP
Dominguez et al. [107]	CNN and transformer architectures	UpRetina database	referable/non-referable AMD, and grade AMD severity scales (no AMD, early AMD, intermediate AMD, and advanced AMD).	Convolutional architectures; AUROC > 95%, κ value = 80%. transformer architectures; AUROC < 75%, and κ < 31%. For referable AMD; F1-score = 92.60%, AUROC = 97.53%, κ = 85.28%. For AMD severity; accuracy = 82.55%, κ = 84.76%.	CFP
Gholami, et al. [108]	Residual network and vision transformer	Kermany dataset, Srinivasan dataset, Li dataset	Normal vs. AMD	ResNet18 accuracy = 94.58%±0.62 on the first dataset, and the ViT encoder = 98.18%±0.55 second dataset, 99.11%±0.39 on the third Li dataset	OCT
Wang et al. [109]	ViT	ODIR-2019	AMD (A), cataract (C), DR (D), glaucoma (G), hypertension (H), myopia (M), other abnormalities (O), and normal (N).	F1 score = 0.932±0.06, AUC = 0.950±0.03.	CFP
Jiang et al. [13]	VGG16, Resnet50, Densenet121, EfficientNet, Vision transformer	Duke University	AAMD, DME, and normal eyes	VGG16 accuracy = 98.51%, Resnet50 = 97.32%, Densenet121 97.02%, EfficientNet = 34.16%,Vision transformer = 99.69%,Vision transformer after pruning = 99.69%	OCT

**Table 7:** Summary of the ML-related studies in AMD diagnosis.

Study	Method	Dataset	Classification	Performance	Modality
Fraccaro et al. [114]	White box (decision trees, logistic regression), Black box (AdaBoost, random forests, SVM)	EHR data from Genoa, Italy	AMD diagnosis	AUC: 0.90–0.92	OCT
Mookiah et al. [12]	Feature extraction (PO, CC), Classification (DT, k-NN, NB, PNN, SVM)	ARIA, STARE datasets	Normal vs. AMD	Accuracy: 91.36–97.78%	CFP
Phan et al. [111]	Color, texture, context analysis, Random forest, SVM	Database of 279 fundus photographs	AMD stage classification	AUC: 0.739–0.874	CFP
Alfahaid et al. [40]	KNN classifier using rotation-invariant uniform local binary pattern texture characteristics	Manchester Royal Eye Hospital	AMD and healthy	Accuracy: 89% (all layers) 89% (superficial) 94% (deep) 98% (outer) 100% (choriocapillaris)	OCTA
Wang et al. [10]	Feature extraction (LCP), Classification (BP, SMO, SVM, LR, NBayes, RF)	OCT dataset from multiple universities	AMD vs. DME vs. healthy macula	Accuracy: 99.3%	OCT
Nugroho et al. [115]	Feature extraction (DenseNet, ResNet50, LBP, HOG), Classification (Logistic regression)	OCT images	Normal vs. DME vs. Drusen vs. CNV	Accuracy: 88–89%	OCT
Hussain et al. [11]	Feature extraction (retinal parameters), Classification (Random Forest)	SD-OCT images	DME vs. AMD	Accuracy: >95%	OCT
Li et al. [23]	Feature integration (RC Net), Classification (RC Net)	OCT images	Eye disease categorization	Accuracy: 99.6%	OCT
Govindaiah et al. [110]	ML and statistical algorithms (Random Forest, Naïve Bayes, Logistic model tree, etc.)	AREDS study data	Late AMD prediction	Accuracy: 72.9%, Sensitivity: 73.8%, Specificity: 72.7%	CFP

**Table 8:** Summary of the retinal experts-related studies in AMD diagnosis.

Study	Method	Dataset	Classification	Performance	Modality
Corvi et al. [43]	Retina specialists	Heidelberg Engineering, Heidelberg, Germany, Carl Zeiss Meditec, Dublin	MNV (AMD)	Specificity = 95.2% and sensitivity = 95.2%	OCTA, OCT, ICGA, FA
Spaide et al. [116]	Ocular pathologists, imaging and image reading center experts, and retina specialists.	OCTA and OCT dataset	MNV (AMD)		OCTA, OCT
Palejwala et al. [117]	Retina specialists	Casey Eye Institute’s retina clinics at Oregon Health and Science University in Portland	CNV (AMD)	Coefficient of variation = 9.4%, pooled coefficient of variation = 5.2%.	OCTA, OCT, FA
Coscas et al. [118]	Retina specialists	The Odeon Ophthalmology Centre in Paris, France.	CNV (AMD)	Accuracy = 94.9%	OCTA, ICGA OCT, FA
de Carlo et al. [119]	Retina specialists	New England Eye Centre patients	CNV (AMD)	Specificity = 91%	OCTA, FA

**Table 9:** Summary of the performance metrics and their description [18,121].

Performance	Equation	Description
Accuracy	$\frac{TP+TN}{TP+FP+FN+TN}$	Shows the proportion of properly predicted occurrences to all of the dataset’s instances
Specificity or FPR	$\frac{TN}{FP+TN}$	Measures the proportion of real negatives to all negatives
Recall or Sensitivity or TPR	$\frac{TP}{TP+FN}$	Measures the proportion of real positives to all true positives
Precision	$\frac{TP}{TP+FP}$	Measures the proportion of all expected positives to true positives.
ROC (Receiver Operating Characteristic)	$\frac{1}{\sqrt{2}}\sqrt{{\mathrm{Sensitivity}}^{2}+{\mathrm{Specificity}}^{2}}$	Illustrates how the genuine positive rate (sensitivity) and false positive rate (specificity − 1) are traded off
IOU (Jaccard similarity index)	$\frac{TP}{TP+FN+FP}$	Intersection Over Union (IOU) is a popular metric for comparing the accuracy of proposed image segmentation to a recognized segmentation
F1-score	$\frac{2\times (\mathrm{Precision}\times \mathrm{Recall})}{\mathrm{Precision}+\mathrm{Recall}}$	A fair assessment of the model’s performance is provided by the harmonic mean of accuracy and recall
Balanced Accuracy (BAC)	$\frac{\left(\mathrm{Recall}\times \mathrm{Specificity}\right)}{2}$	Average of sensitivity and specificity, providing an overall measure of classification performance
AUC (Area under the ROC Curve)	-	AUC provides an aggregate measure of performance across all possible classification thresholds
DSC (Dice similarity coefficient)	$\frac{2TP}{2TP+FP+FN}$	Values in the range [0,1], 0 indicating no spatial overlap, 1 indicating complete overlap

## Data Availability

The datasets used and analyzed during the current study are available from the corresponding author upon reasonable request.
